# Probabilistic Machine Learning Estimation of Ocean Mixed Layer Depth From Dense Satellite and Sparse In Situ Observations

**DOI:** 10.1029/2021MS002474

**Published:** 2021-11-30

**Authors:** Dallas Foster, David John Gagne, Daniel B. Whitt

**Affiliations:** ^1^ Department of Aeronautics and Astronautics Massachusetts Institute of Technology Cambridge MA USA; ^2^ National Center for Atmospheric Research Boulder CO USA; ^3^ NASA Ames Research Center Moffett Field CA USA

**Keywords:** machine learning, satellite, ocean, mixed layer, Argo, uncertainty quantification

## Abstract

The ocean mixed layer plays an important role in the coupling between the upper ocean and atmosphere across a wide range of time scales. Estimation of the variability of the ocean mixed layer is therefore important for atmosphere‐ocean prediction and analysis. The increasing coverage of in situ Argo profile data allows for an increasingly accurate analysis of the mixed layer depth (MLD) variability associated with deviations from the seasonal climatology. However, sampling rates are not sufficient to fully resolve subseasonal (<90 day) MLD variability. Yet, many multivariate observations‐based analyses include implicit modeled subseasonal MLD variability. One analysis method is optimal interpolation of in situ data, but the interior analysis can be improved by leveraging surface data with regression or variational approaches. Here, we demonstrate how machine learning methods and satellite sea surface temperature, salinity, and height facilitate MLD estimation in a pilot study of two regions: the mid‐latitude southern Indian and the eastern equatorial Pacific Oceans. We construct multiple machine learning architectures to produce weekly 1/2° gridded MLD anomaly fields (relative to a monthly climatology) with uncertainty estimates. We test multiple traditional and probabilistic machine learning techniques to compare both accuracy and probabilistic calibration. We validate our methodology by applying it to ocean model simulations. We find that incorporating sea surface data through a machine learning model improves the performance of spatiotemporal MLD variability estimation compared to optimal interpolation of Argo observations alone. These preliminary results are a promising first step for the application of machine learning to MLD prediction.

## Key Points

- Machine learning models using both ocean profile and surface data improve mixed layer depth variability estimates over models using profiles alone
- Model performance is dependent on spatial location and strength of the sub‐seasonal variance
- Probabilistic parametric techniques capture uncertainty better than sampling or ensemble approaches

## 1 Introduction

The ocean surface mixed layer depth (MLD) is an important factor in atmospheric and oceanic dynamics. For example, the MLD modulates sea‐surface temperature dynamics (Deser et al., 2010), air‐sea interaction (Frankignoul & Hasselmann, 1977; Kraus & Turner, 1967), ocean water mass formation and properties, as well as ocean circulation (Hanawa & Talley, 2001; Stommel, 1979). While there have been several recent efforts to observe and quantify the global climatological seasonal cycle of the MLD based on the in situ array of thousands of vertically profiling Argo floats (Holte et al., 2017; Schmidtko et al., 2013; Whitt et al., 2019), little effort has been devoted to quantifying the aseasonal (non‐climatological) variability of the MLD because the Argo array is not sufficiently large to fully resolve subseasonal (<90 days) MLD variability. In this study, we take a preliminary step toward improved observational estimates of aseasonal MLD variability by identifying and leveraging relationships between the MLD and sea surface salinity (SSS), temperature (SST), and height anomalies (SSH) in a machine learning framework.

Our approach is predicated on the hypothesis that there are significant relationships to be learned between the MLD and the ocean surface variables, including SST, SSS, and SSH. This hypothesis is motivated by previous studies that have identified and leveraged ocean surface‐to‐interior relationships (Gaube et al., 2019; Guinehut et al., 2012; Helber et al., 2013; Isern‐Fontanet et al., 2008), including for MLD estimation (Buongiorno Nardelli et al., 2017). There are many physical reasons why the MLD might be related to surface variables. For example, depression or shoaling of the thermocline that manifests in sea level anomalies may also facilitate changes in MLD by reducing or increasing the density near the mixed layer base and hence changing the MLD. Likewise, an increase in the surface density (due to changes in SST or SSS associated with air‐sea fluxes, for example) can decrease the near‐surface stratification and hence increase the MLD. Or, horizontal circulation anomalies may introduce relationships among anomalies in MLD, SST, SSS, and SSH due to horizontal advection of the whole upper‐ocean water column. However, the ocean surface variables are also thought to vary for reasons unrelated to MLD variability (and vice versa), and the physics of the relationships between the ocean surface variables and the MLD are complex and vary both spatially and temporally. Based on prior work, we expect that atmospheric variables, such as wind, may provide information about the ocean MLD that is independent of the ocean surface variables. For example, the wind speed can sometimes explain the transient mixed‐layer deepening during a storm (Pollard et al., 1973; Price et al., 1978). More generally, atmospheric variability can explain some of the temporal variability in the MLD on a wide range of timescales in many ocean regions (Alexander et al., 2000; Carranza & Gille, 2015; Large et al., 1986; Martin, 1985; Waniek, 2003; Whitt et al., 2019; Zhou et al., 2018). But, some fraction of the atmospherically driven MLD variability also manifests in the ocean surface variables. For example, wind‐driven entrainment can change the SST and SSS (Large et al., 1986; Price et al., 1978), and the response timescale of the SST and SSS to atmospherically driven surface flux anomalies is sensitive to the MLD (Frankignoul & Hasselmann, 1977). Ultimately, it remains an open question whether, to what degree, and on what space and timescales surface salinity, temperature and height provide predictive information about the MLD, particularly on subseasonal and shorter timescales (<90 days) and mesoscale and smaller spatial scales (<500 km). Hence, work is required to fully understand the physical basis and create predictive models and historical analyses of MLD variability that optimally leverage all available observations. This study takes a step toward the latter end by using machine learning in an attempt to identify and leverage relationships between ocean surface variables and MLD for prediction and analysis.

Due largely to the increasing coverage of the Argo array (Holte et al., 2017), the MLD is increasingly well‐observed globally. Despite this improvement, however, the data is insufficient to recover MLD variability on the spatial or temporal scale of state‐of‐the‐art global ocean models. For example, the CESM POP2 ocean model (whose data we use in one of our experiments, see Section 4.1) uses a 1/10° resolution grid, runs with a time step of about 3 min, and outputs data averaged over 5‐day intervals. The coarsest gridded satellite product (salinity) used in this study is available every 7 days on a 1/2° resolution global grid; the satellite samples at least once every (roughly) 20,000 square kilometers every 7 days (Le Vine et al., 2007). Meanwhile, for the Argo data set used in this manuscript, there is, on average for a given 7‐day period, 1 Argo profile per (roughly) 150,000 square kilometers. Furthermore, the Argo profiles are not equidistant and are often spatially clustered.

Modern attempts to recover variables using a hybrid data collection of in situ and satellite data typically use optimal interpolation (Cabanes et al., 2013; Guinehut et al., 2012; Roemmich & Gilson, 2009), or a data assimilation reanalysis using ocean models (Balmaseda et al., 2015; Buongiorno Nardelli et al., 2017; Cummings & Smedstad, 2013; Helber et al., 2013; Masina et al., 2017). While the use of optimal interpolation and data assimilation can create accurate, fine resolution gridded MLD products, the methodology can introduce biases and artifacts derived from the assimilation of the ocean models that are not inherent in the data. Our aim in this study is to demonstrate the utility of informing MLD estimation using satellite surface data through a purely observation‐based machine learning framework. Therefore, we test the possibility of constructing a data‐driven relationship between sea surface variables and the MLD. The results of this study serve as a preliminary step that justifies further extension of the methodology and framework to eventually include a machine learning‐based global reanalysis of the MLD that can be evaluated against state‐of‐the‐art data assimilation products and, potentially, be included in data assimilation reanalysis schemes.

The application of machine learning to the geosciences is a rapidly growing field (Irrgang et al., 2020; Lary et al., 2016; Monteleoni et al., 2013; Reichstein et al., 2019; Weyn et al., 2019). The machine learning approach offers a flexible, data‐driven route to regression and classification tasks that has been used for parameterizations (Bolton & Zanna, 2019; Brenowitz & Bretherton, 2018; Gagne et al., 2020; Gentine et al., 2018; Jiang et al., 2018; O'Gorman & Dwyer, 2018; Rasp et al., 2018), forecasting (Hsieh & Tang, 1998; Irrgang et al., 2020; McGovern et al., 2017; Pathak et al., 2018; Ukkonen & Mäkelä, 2019; Weyn et al., 2019), data assimilation (R. Cintra et al., 2016; R. S. Cintra & de Campos Velho, 2018; Wahle et al., 2015), and remote sensing (Lary et al., 2016; Ouali et al., 2017). Unfortunately, many successes in machine learning research are also in over‐determined regimes, in which the amount of data is large in comparison to the number of independent parameters. Extrapolation regimes, where data are sparse in one or more dimensions, are known to be problematic because the prediction depends more heavily on the underlying assumptions of the model. This is particularly problematic in oceanography, where many unknown quantities are two or three dimensional, and data availability is still relatively sparse.

While the study of machine learning can trace its history to Rosenblatt's perceptron (Rosenblatt, 1958), the implementation of early machine learning methods and architectures in a data‐driven way was considered computationally infeasible for moderate to large applications until the late 1980s with the development of the back‐propagation algorithm (Rumelhart et al., 1986), which enabled training of multi‐layered neural networks. Despite advances through the nineties and early twenty‐first century, the deep learning revolution did not occur until 2006 (Goodfellow et al., 2016) when an explosion of reliable training data, computing power, neural network layers, and regularization techniques have dramatically increased neural network accuracy. As demonstrated in Guo et al. (2017), this improvement in accuracy has also hindered the capacity of neural networks to be well‐calibrated, that is, when forecast probabilities match the system's true probabilities, and hence offer accurate representations of the underlying probability distributions. The ability for a neural network to be well‐calibrated is of critical importance. Data assimilation research has repeatedly shown that proper estimation of the background error covariance can improve reconstruction estimates (Valler et al., 2019). In the estimation of sea surface temperature or sea level anomaly, mis‐quantification of atmospheric uncertainties has also been shown to cause significant and non‐local errors in reanalysis estimates (Chaudhuri et al., 2016). Parallel developments have led to the field of probabilistic neural networks to address this calibration problem in machine learning.

The ultimate goal of probabilistic neural networks is to be able to accurately and precisely define the posterior probability distribution conditioned on the data. Using a Bayesian framework allows us to easily account for sources of error and randomness in the data, weights, or model. The gold standard for this task is often sampling from the posterior distribution using a Markov Chain Monte Carlo (MCMC) scheme (Brooks, 2011; Gelman et al., 2013), but this approach is still computationally infeasible for modern neural networks. There have been several approximations and techniques developed for producing estimates of the posterior probability including the development of Bayesian Neural Networks, with weight uncertainty (Blundell et al., 2015; Neal, 1996), Stochastic Gradient Langevin Dynamics (Welling & Teh, 2011), Variational Inference (Hoffman & Blei, 2015; Kingma et al., 2015; Paisley et al., 2012), Probabilistic Backpropagation (Hernández‐Lobato & Adams, 2015; Rezende et al., 2014), Dropout (Ba & Frey, 2013; Gal & Ghahramani, 2016; Gal et al., 2017; Hinton et al., 2012; Maeda, 2014), Variational Autoencoders (Kingma & Welling, 2014), and Deep Ensembles (Lakshminarayanan et al., 2017).

Despite the numerous techniques to inject uncertainty estimates into machine learning, the performance of any approach is still underwhelming. Recent arguments have been made that ensembles of techniques outperform any one approach (Dormann, 2020; Guo et al., 2017; Kuleshov et al., 2018; Lakshminarayanan et al., 2017; Nixon et al., 2019). Due to the complex nature of the analytical posterior distributions, lack of complete data, prohibitive cost of training, and sensitivity to the nature of the application, an understanding of which methodology is appropriate is still in its infancy. Recently there has been some research comparing popular uncertainty quantification techniques in Deep Learning (Ashukha et al., 2020; Caldeira & Nord, 2020; Labach et al., 2019; Lakshminarayanan et al., 2017). Unfortunately, it still remains an open question as to how these methods perform in the geosciences (where probabilities are often non‐Gaussian, non‐trivial, non‐stationary, and high‐dimensional) and what the best practices might be. This study serves as a step into answering this question by testing various probabilistic machine learning methods used for high‐dimensional data with both Gaussian and non‐Gaussian distributions on MLD estimation, which serves as an example problem in this respect.

Our goals for this manuscript are two‐fold. For our first goal, we investigate to what extent the aseasonal variability in SSS, SST, and SSH are related to, and thus useful for estimating, the aseasonal variability of the MLD. In particular, we study two geographic regions, (a) the eastern equatorial Pacific Ocean from 10°S–10°N to 150°–120°W and (b) the southern Indian Ocean from 45°–35°S to 55°−115°E, over the 2011–2015 time period. As detailed in Section 2, these regions are useful test cases because both are characterized by at least modest subseasonal MLD variability (>10 m subseasonal standard deviations), but the magnitudes of subseasonal variability, the climatological annual cycle, and interannual variability all differ substantially (Whitt et al., 2019). Thus, the two regions reflect useful and distinct test cases for evaluating machine learning model performance. Our analysis takes two stages. We first train a series of neural network architectures on the CESM POP2 ocean model data interpolated to the same grid as the satellite observation data. This allows us to pre‐train machine learning models using complete MLD maps and provides a proof of concept for our scientific approach. From these machine learning models we can also understand the extent to which each of the input variables impacts the MLD predictions. In the second stage we perform transfer learning by reusing these pre‐trained model weights as a starting point for the training of neural network architectures to produce gridded MLD estimates using the satellite observational surface variables as inputs and evaluate model performance at the Argo MLD observation locations. We compare the machine learning approaches, which only use surface values as inputs, to the traditional optimal‐interpolation technique that estimates using the actual MLD values from the in situ Argo profiles. The differences in performance between the machine learning methods and optimal‐interpolation schemes will reveal the extent to which the sea surface variables are useful in predicting spatiotemporal variability in the MLD. If successful, this methodology can produce MLD maps derived from satellite SST, SSS, and SSH data to supplement and assimilate with the sparse in situ data.

For our second goal, we focus on understanding the probability distribution of the MLD that is learned by the neural network. As a first step, we evaluate how well calibrated the neural network estimates are and what spatial and temporal patterns are revealed through sampling these distributions. We choose three probabilistic machine learning methods that cover two distinct types of uncertainty quantification: parameterization‐ and sampling‐based methods. By evaluating these methods, we aim to understand the appropriateness of a Gaussian distribution to the data and the ability for sampling machine learning methods in exploring the posterior distribution. Finally, we compare the machine learning uncertainty quantification against uncertainty estimates from the optimal‐interpolation approach. As before, this last comparison will reveal the extent to which the sea surface variables inform us about the uncertainty in the MLD.

These methods are certainly not exhaustive and so this study is a first step to a better understanding of the predictability of the aseasonal MLD variability given the dynamics of some sea surface fields, and how machine learning can be used as a tool in this investigation. The outline of the body of the study is as follows: first, in Section 2 we detail the data and describe the data processing and methodology; second, in Section 3 we describe the mathematical framework and relevant machine learning architectures that we implement; lastly, in Section 4 we explain and detail the experiments and results.

## 2 Data

### 2.1 CESM POP2 Ocean Model Data

For the ocean model set of experiments we utilize data from the CESM POP2 model in a hindcast forced by JRA55‐do (Tsujino et al., 2018) atmospheric reanalysis from 1958 to 2006 and initialized with an oceanic climatology as in, for example, Deppenmeier et al. (2021). The model outputs include the ocean MLD, SSS, SST, and SSH time averaged every 5‐days and an approximate latitude and longitude resolution of 0.1° from 1983 to 2006.

There is significant seasonal variability in SSS, SST, SSH, and MLD that must be carefully removed in order to better analyze the aseasonal variability and relationships between these variables. In particular, we extract data from one decade 1989–1998 and divide this period based on the Multivariate ENSO Index v2 values (Zhang et al., 2019) to ensure even division of phases and magnitudes of ENSO activity across the climatology and anomaly data sets. Among the anomaly subset, we further split the data into training/testing and validation subsets roughly according to the ENSO index. We use the years of 1989, 1991, 1992, 1993, and 1994 to compute a climatology for each of these variables by computing a binned monthly mean and standard deviation on 4 weeks boxcar moving averages of the data over this time period. These operations sufficiently smooth out aseasonal variability to create the monthly climatologies. These climatologies are then used to compute regular and standardized anomalies for data from the years 1995–1998.

Taking the anomalies (1995–1998), for each of the regions of interest, we down‐sample by linearly interpolating (without any spatial smoothing) onto a grid with 0.5° spacing in order to match the satellite observation grid used in the Argo experiments. In addition, we carefully split the data in an effort to avoid contamination of the validation results of the machine learning models because of the autocorrelation and non‐stationarity inherent in the data. By ensuring the training, testing, and validation data are sufficiently different we can also implement methods to minimize overfitting. Data starting from January 21, 1998 through the rest of 1998, which features strong positive and negative ENSO index values, is taken as validation data—a total of 70 5‐day periods (data is temporally averaged over the 5‐day period) ‐ for which the machine learning model results will be presented. The training data and test data is taken randomly from 1995, 1996, 1997, up to and including January 16, 1998 for which 200 5‐day periods are reserved for training data and 30 5‐day periods are used for internal testing. To retain separation of the validation data from the training data, half of the test data comes from the last 15 5‐day periods of the train/test split timeline while the other 15 are randomly sampled from the remaining distribution. This split design creates a buffer of 70 days between the last training period and the first validation period. The results of the machine learning models on this data set are presented in Section 4.1.

### 2.2 Satellite Optimal Interpolation and Argo Data

In this study we consider the use of optimally interpolated satellite products of SSS, SST, and SSH. While the processing of these products from raw satellite data may introduce bias or uncertainty, the processing in these standard products includes important error corrections and calibrations in addition to interpolation and has been thoroughly vetted and validated. Validation of the gridded products suggest they accurately resolve wavelengths down to about 300 km on a weekly basis over most of the globe (Lambin et al., 2010; Le Vine et al., 2007; Melnichenko et al., 2016; Systems, 2017; Zlotnicki et al., 2019). That is, the representation of variability on the grid starts to degrade (for various reasons and to different degrees for different variables) at wavelengths of about 300 km, which is about 3‐times longer than the smallest resolvable wavelength on the grid. While the use of optimally interpolated satellite data should be considered as a source of additional uncertainty in the evaluation of our results, we believe that the use of these products presents a realistic use case for possible practitioners or users of this methodology.

#### 2.2.1 Sea Surface Salinity

SSS data is the analysis of Melnichenko et al. (2016), which is an optimal interpolation of observations from the Aquarius satellite sensor (Le Vine et al., 2007) and uses corrections to minimize bias relative to in situ data. The data exists on a 0.5° grid, temporally averaged over 7‐day weeks, spanning roughly 2011–2015 (200 weeks). As this SSS product is the most time‐limited of the surface data, it defines the time period of our study. A random 150 weeks sample constitutes the training data, with the remaining being used for testing and validation. This grid is the coarsest of all the variables and thus will form the basis from which we interpolate and re‐sample the other data onto. To calculate an estimate of the climatology, we calculate monthly means using only the training data, taking a 4 weeks boxcar moving average, binning data into months and averaging over the bins.

Training and testing data are randomly sampled from 2011 through to the end of 2014, with 150 weeks reserved for training, 25 reserved for testing. The validation and testing data sets need to be sufficiently separated from the training data set in order to ensure the effect of temporal autocorrelation in the data does not leak into the validation results. As a consequence, the validation data comprise the last 25 weeks of the Aquarius data in the first half of 2015.

#### 2.2.2 Sea Surface Temperature

SST data comes from the GHRSST Level 4 Global Foundation Sea Surface Temperature analysis data set (Systems, 2017). This data set uses Optimal Interpolation from several microwave sensors. The data exists on a $\frac{1}{4}$ degree, daily grid spanning roughly 2001–2018. To calculate an estimate of the climatology, we set aside the years 2011–2015 and calculate a 4 weeks boxcar moving average on the remaining data. From the smoothed data, we take bins according to each month and average over the bins, resulting in an approximate monthly climatology to which we interpolate to a weekly resolution. To calculate anomalies, we bin the 2011–2015 data into months and subtract the (interpolated) monthly climatology. Then, to be able to compare to the salinity data set, we down‐sample from the daily values to weekly data and optimally interpolate onto a $\frac{1}{2}$ degree grid. The SST anomalies from 2011 to 2015 are split into training, testing and validation subsets in exactly the same way as the SSS anomalies for consistency.

#### 2.2.3 Sea Height Anomaly

SSH data comes from the MEaSUREs Gridded Sea Surface Height Anomalies data set (Zlotnicki et al., 2019). The data exists on a $\frac{1}{6}$ degree grid nominally as 5‐day averages spanning roughly 1992–2019. We do not calculate and remove climatologies from this data set. To be able to compare to the salinity data set, we down‐sample from 5‐ to 7‐day weekly averages and optimally interpolate onto a $\frac{1}{2}$ degree grid. Finally, the SSH anomalies from 2011 to 2015 are split into training, testing and validation subsets in exactly the same way as the SSS and SST for consistency.

**Figure 1:**
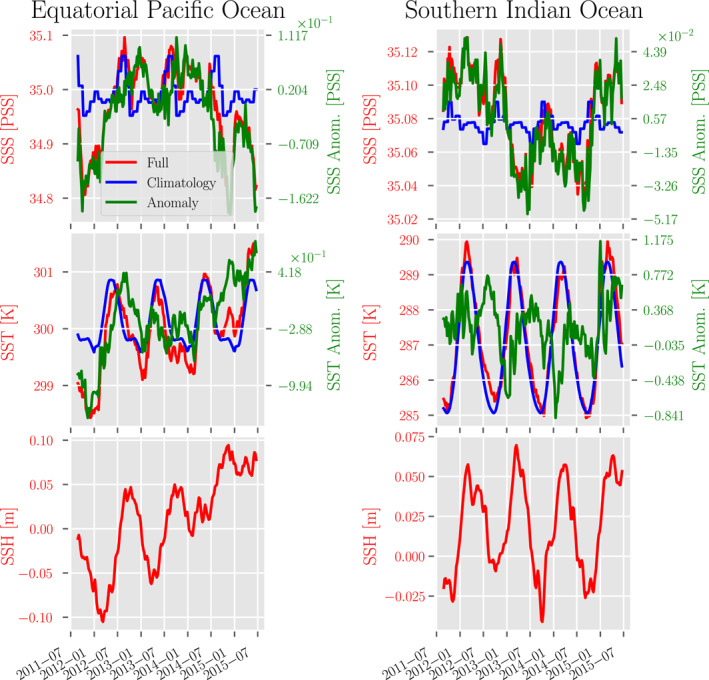
Several time series of the spatially averaged SSS, SST, and SSH in each region at weekly resolution in the (left) equatorial Pacific (120°W, 10°S)–(150°W, 10°N) and (right) southern Indian Ocean (45°S, 55°E)–(35°S, 115°E) regions. The time series include the spatially averaged (red) satellite data, (blue) estimated climatology, and (green) resulting anomaly.

Figure 1 shows the time series for each of the aforementioned satellite input data, along with the corresponding calculated climatologies and anomalies, spatially averaged over the regions of interest in this manuscript—the equatorial Pacific Ocean (EPO) and southern Indian Ocean (SIO) regions.

### 2.3 Argo Mixed Layer Depth

Argo data is available through Cabanes et al. (2013). The MLD is defined for about 1.5 million profiles of temperature and salinity that pass quality controls in the time span from 2000 to 2017 (Whitt et al., 2019, 2020). We adopt the definition used in Whitt et al. (2019) and Large et al. (1997) and use it throughout this study (in both models and observations). In a given profile, the MLD is defined to be the first depth at which the local vertical buoyancy gradient exceeds the maximum average vertical buoyancy gradient from the surface to depth (see Whitt et al. [2019] for details). But, there are multiple definitions of the mixed layer depth in common use (some comparisons between our chosen definition and other common definitions are presented in Whitt et al. [2019]). Our choice of MLD definition thus represents an additional source of model error that we do not account for, but could be studied by analyzing the impact of various definitions through the methodology considered here.

**Figure 2:**
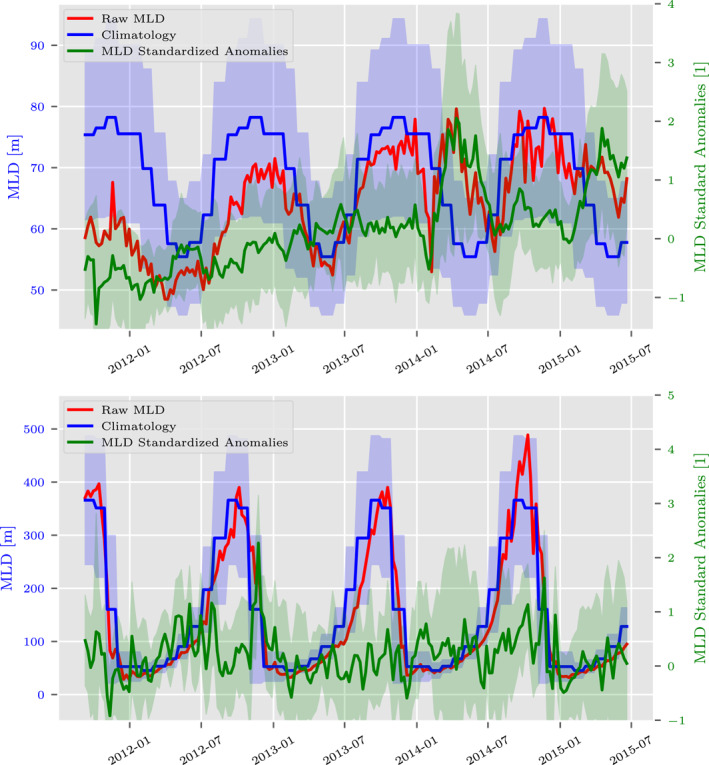
Several time series of the average mixed layer depth (MLD) in each region at weekly resolution in the (top) equatorial Pacific and (bottom) southern Indian Ocean, including the ensemble average of the MLD profiles over the domain (red), the ensemble average of the corresponding standardized MLD anomalies (green), and the area‐average of the gridded monthly MLD climatology (blue). The blue shading represents the area‐average of the gridded monthly standard deviations, and the green shading represents the ensemble standard deviation of the profile‐wise standard anomalies.

To calculate an estimate of the climatology from the individual MLD measurements, we take the years 2002–2010, and 2016–2017, bin the data into 2° latitude and 4° longitude bins, re‐sample onto a daily grid and take 4 week moving averages in each bin. This smoothed data is then grouped into months. Both an average and standard deviation are calculated for the monthly climatologies (in each bin). The choice of bin size is ad‐hoc but determined from the smallest bin size to ensure that there is sufficient data available, that is, at least 4 profiles per month, to calculate monthly statistics and climatologies for the areas of interest (There are small or isolated regions that do not have sufficient data, but this does not impact our analysis). Anomalies are created by taking each profile from the withheld 2011–2015 Argo data and subtracting the climatology according to the profile's bin and date. In addition, for each profile, we divide by the bin's corresponding monthly standard deviations to create standardized anomalies. Figure 2 shows the time series of the raw MLD data, including the ensemble average of the individual profiles in each region, the ensemble average of the standardized anomalies at each profile, and the area‐average of the gridded climatology, in two spatial regions under study (120°W, 10°S)–(150°W, 10°N) and (45°S, 55°E)–(35°S, 115°E). The character of the anomalies and standardized anomalies are not dissimilar, but the standardized anomalies have a more appropriate scale for machine learning purposes (see the Acknowledgments for data availability).

### 2.4 Evaluation Regions

We are interested in understanding how the performance of the machine learning models are dependent upon the variability of the MLD. In order to evaluate this dependence, we explore two oceanic regions that exhibit different subseasonal and interannual MLD variability. First, we choose the equatorial Pacific Ocean (EPO) (10°S–10°N and 150°–120°W), which has modest subseasonal MLD standard deviations (∼15 m), a small climatological annual cycle (∼20 m), and substantial interannual variability (see Figure 2 and Whitt et al. [2019]). Second, we choose to study the southern Indian Ocean (SIO) (45°–35°S and 55°–115°E), which features larger subseasonal standard deviations (∼50 m), a much larger climatological annual cycle (∼300 m), but relatively weak interannual variability. Both regions contain substantial subseasonal MLD variability to learn, but the absolute magnitudes of the subseasonal variability as well as the relative magnitudes of subseasonal, seasonal, and interannual variability differ dramatically.

**Figure 3:**
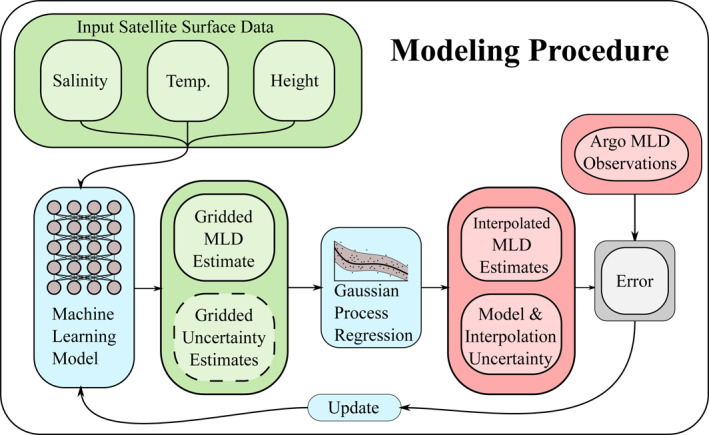
A schematic of the Argo data set experiment modeling procedure. Satellite sea surface data is fed into the machine learning model to produce a gridded mixed layer depth (MLD) estimate (with some form of an uncertainty estimate if the machine learning model is probabilistic). To compare with the observations and optimize parameters, these gridded estimates are fed into a Gaussian process regression model (with its own hyper‐parameters that are optimized) to produce MLD estimates interpolated to the locations where the Argo observations exist. These interpolated estimates are automatically associated with uncertainty estimates that are derived from either just the Gaussian process interpolation uncertainty (if the model is deterministic) or a combination of the Gaussian process uncertainty with ML model uncertainty (if the ML model has uncertainty estimates). The interpolated estimates are then compared with the observations to estimate various errors. The CESM POP2 ocean model data set experiments have the same modeling design but do not require interpolation, since ocean model MLD is on the same grid as the inputs.

In order to test our observations‐based framework for estimating MLD using sea surface information we perform the following experiment on each region of interest. On the 150 (out of 200 total) weeks of training data, which are separated as described in Section 2.2.1, we apply the training procedure summarized in Figure 3 and described in more detail in Section 3 (see Acknowledgments for a link to the software).

On the remaining 50 weeks of testing and validation data the model predicts a dense grid of MLD estimates based solely on the sea surface information as input. From this dense grid, we interpolate the estimates onto the locations where in situ Argo profile observations of the MLD exist and compute error statistics between the interpolated estimates and the observations. The interpolation is done using a Gaussian process (see Appendix C) regardless of the machine learning method. We denote this testing procedure as measuring the out‐of‐sample performance of the method.

## 3 Methods

We consider a simple but general model for the relationship between the surface variables, salinity (SSS), temperature (SST), and height (SSH), and mixed layer depth model output (*d*),

(1) d=f(SSS,SST,SSH;θ)+σ,σ∼N(0,Σ).

where *θ* refers to the collection of function parameters (we apologize for any confusion with the notation for potential temperature, which we do not consider in this text). The surface variables exist on a pre‐specified grid, **x**, of total size *M* and the function *f* may generally couple surface variables from across this grid to produce *d* at a particular grid point. The difference between the mixed layer and the output of *f*, *σ*, is assumed to be a normally distributed random variable according to the covariance *Σ* that expresses the spatial uncertainties in this functional relationship. The exact structures and parameterizations of *f* that we use in this study are described in Section 3.1 while the methods we use to specify *Σ* are presented in section Appendix A.

Both the functional relationship *f* and the covariance matrix *Σ* are data‐driven (i.e., agnostic to the underlying physics) and informed via observations *d*_*o*_. For the CESM POP2 ocean model experiments we have access to these “observations” at each grid point where we produce MLD estimates. For the Argo data set experiments, the observations *d*_*o*_ exist at arbitrary (ungridded) locations, **x**_*o*_ where freely drifting Argo floats collect a profile. In order to couple the gridded surface variables with the ungridded in situ MLD observations, we define the relationship between our model and the observations to be a Gaussian process,

(2) $$d_{o}=Ld+\nu ,\;\nu ∼N\left(0,V\right),$$

which is defined and detailed in Appendix C. Importantly, *L* and *V*, the spatial projection and covariance matrices, are independent of the observation values and only depend on the observation locations, model grid locations, and model uncertainties. The Gaussian process relationship, in our study, is entirely a spatial relationship that accounts for spatial covariance between observations of the MLD. This implicitly means, however, that *L* and *V* change depending on the particular week the data is from, but only because the particular locations **x**_*o*_ where estimation and validation occurs vary from week to week.

A further consequence of the chosen relations between the observations and model (Equations 1 and 2) (i.e., the definitions of *ν* and *σ*) is the objective function, that is, the conditional likelihood probability distribution, that will be maximized to fit the parameters of the nonlinear function *f*:

(3) $$\ln p\left(d_{o}|d\right)=-\frac{1}{2}{\left(d_{o}-Ld\right)}^{T}V^{-1}\left(d_{o}-Ld\right)-\frac{1}{2}\ln|V|-\frac{m_{o}}{2}\ln2\pi .$$

where |*V*| is the matrix determinant of *V* and *m*_*o*_ is the number of individual observations, or entries of *d*_*o*_. For the CESM POP2 ocean model data set, there is no need for a Gaussian process regression interpolation model to translate between model and observation space and the corresponding likelihood is simply

(4) $$\ln p\left(d_{o}|d\right)=-\frac{1}{2}{\left(d_{o}-d\right)}^{T}\Sigma^{-1}\left(d_{o}-d\right)-\frac{1}{2}\ln|\Sigma |-\frac{M}{2}\ln2\pi .$$

and *M* is the number of grid points.

Details of this optimization procedure are given in Section 3.1. Here, it is implicitly understood that *d*, and hence *p*(*d*_*o*_|*d*), is a function of the input variables SSS, SST, SSH, the architecture of the function *f*, and the parameters of *f*, *θ*.

The Gaussian assumptions made in Equation 1 is primarily for notational convenience. The model definition (Equation 1) can easily be modified to include non‐Gaussian noise by including a stochastic component in *f*, *f*(SSS, SST, SSH; *θ*, *σ*). This type of noise component is important if we expect the noise to be a nonlinear function of the surface variables. To account for this possibility, two of the probabilistic machine learning methods that we test in this study, Dropout and Variational Auto‐Encoders (see Appendix A) are formally of this type and require sampling to determine the covariance for use in the Gaussian process. The Gaussian assumption made in Equation 2 is a reflection of the belief that the interpolating operator between the gridded locations and Argo locations is appropriately approximated by a linear function. We believe that this is not overly restrictive since most optimal interpolation techniques make similar assumptions.

### 3.1 Machine Learning

The main objective of this study is to learn a relationship between the sea surface variables (SSS, SST, SSH) and MLD. Without an a priori physics‐based model, one must choose a reasonably parameterized model to approximate this relationship. Traditionally this relationship is represented via some linear or simple nonlinear parameterization where one hopes that the true relationship lies in, or is not too far from, the output space of the model. For example, a basic linear model that we test in this study is of the form,

(5) $$d_{\ell }=[\begin{array}{c} c_{1}\left(x\right)\\ c_{2}\left(x\right)\\ c_{3}\left(x\right) \end{array}]\cdot [\begin{array}{c} \text{SSS}\\ \text{SST}\\ \text{SSH} \end{array}]+b+\sigma ,\;\sigma ∼N\left(0,\Sigma \right)$$

Such models, however, are typically not expressive enough to represent arbitrary relationships. The revolution of machine learning, and, in particular, deep learning, has been borne out of the need to express arbitrary functional relationships amid a dearth of observational data. One of the quintessential deep learning models is the feedforward neural network (FNN), or artificial neural network (ANN) (Goodfellow et al., 2016). ANNs are represented by composing together many different functions in series to form a chain,

(6) $$f\left(x\right)=f^{\left(n\right)}\left(f^{\left(n-1\right)}\left(\cdots f^{\left(1\right)}\left(x\right)\cdots \;\right)\right),$$

(7) $$f^{\left(i\right)}\left(x\right)=a(x^{T}W_{i}+b_{i}),$$

where *W*_*i*_ is a matrix of weights, *b*_*i*_ is a bias term, and *a*(⋅) is what is referred to as an “activation function,” that applies a simple non‐linearity element‐wise to the affine transformation of the input, *x*. Common examples of activation functions include the sigmoid, softplus, and families of rectified linear functions. Based on the experiments in Gal (2016), we implement the (leaky) rectified linear unit as the activation function in all of our neural network layers, although it is possible that, among all of the available activation functions, another function would result in superior performance.

FNNs represent dense interactions between inputs, which requires an increasingly large number of resources for large input dimensions. For 2D and 3D data sets that have a notion of locality, such as images, there are more efficient neural networks that take advantage of spatial structures inherent in the data. Convolutional Neural Networks (CNNs) are a specialized type of architectures that utilizes a convolution operator in some layers. This convolution operator introduces kernel matrices that perform a sliding weighted sum over the input image to produce corresponding filters. See Goodfellow et al. (2016) for a complete guide. Note that the same type of activation functions are also commonly used in CNN architectures. For a review of the specific architectures used in this work, see the appendix. Because of our use of 2D input data, we primarily make use of this (CNN) style of neural network architecture in this work. We will denote the collection of neural network parameters as *θ* = {*W*_1_, *…*, *W*_*n*_, *b*_1_, *…*, *b*_*n*_}.

### 3.2 Training

The training of a neural network entails obtaining an estimate of the parameters, $\hat{\theta }$, and hence the model outputs $\hat{d}$, by approximately solving the optimization problem,

(8) $$\begin{array}{ccc} \hat{\theta } & = & \underset{\theta }{\text{arg max}}\ln p\left(d_{o}|d\right)\\ & = & \underset{\theta }{\text{arg min}}\{g\left(\theta \right)-\sum_{j=1}^{n_{\text{train}}}\ln p_{j}\left(d_{o}|d\right)\} \end{array}$$

where *g*(*θ*) is a regularization function that is applied to both constrain the possible parameter values and stabilize the optimization procedure. As written, *p*_*j*_(*d*_*o*_|*d*) refers to the joint probability distribution between the *j*th input and output data. The optimization procedure includes all training data but, in practice, subsetting is common (as in batch gradient descent [Ruder, 2016]). We only seek an approximate solution to Equation 8 for two reasons: first, the optimization problem is highly non trivial, non‐convex, and high‐dimensional with many local minima and obtaining a global minimum is infeasible; second, the ultimate goal is for the parameters to lead to a function *f* that generalizes well to data not in the training set and over‐training might ultimately hinder this goal (Caruana et al., 2001). The problem of over‐fitting and poor generalization is one of the largest obstacles to good machine learning performance, particularly in applications where prediction involves extrapolation beyond whatever data was in the training set. All of the neural networks implemented for this study are done using the TensorFlow and TensorFlow Probability frameworks (Abadi et al., 2016; Dillon et al., 2017).

For the Argo data set experiments, because our study is limited to only 150 training weeks, we implement a non‐standard training strategy to help reduce overfitting. For each epoch (a single run through the entire training data) we divide the 150 training weeks randomly into 6 batches of 25 weeks. The first batch is held out and the current loss on that batch is saved. For each subsequent batch, the loss for that batch is used to update the model parameters. To update the parameters, we use the Adam optimizer with initial learning parameter set to 0.001 (Kingma & Ba, 2015). With the updated model parameters, we calculate a new loss on the first, held‐out batch. If that new loss is less than the saved loss, then the updated parameters are accepted and the new loss is saved. If the new loss is larger than the saved loss then the parameters are only accepted with

probabilityofacceptance=exp(savedloss−finalloss).

This training strategy reduces the amount of overfitting because it forces updates to be generalizable to the held out batch, which acts as a “testing batch.”

FNNs with enough hidden layers have been proven to serve as a universal approximator (Cybenko, 1989; Hornik et al., 1989; Leshno et al., 1993). This means that, at least theoretically, there exists a FNN that can represent whatever functional relationship exists between the sea surface variables and MLD. Unfortunately, there is no guaranteed way to find this optimal relationship. While the optimization problem (Equation 8) has a natural inherited probabilistic framework, even an exact solution has no guarantee of agreeing with the “true” relationship. The construction of these optimization frameworks and the regularization functions is often done by trial and error since there is, as of yet, no clear causal relationship between tuning the architecture settings and the resulting uncertainty estimate—even if the model can be viewed through a (Bayesian) probabilistic framework.

Finally, since the (approximate) solution to Equation 8 is not accompanied with natural uncertainty estimates for the parameters, it can be difficult to obtain calibrated probabilistic estimates of $\hat{d}$. To truly obtain samples from the posterior *p*(*d*|*d*_*o*_, SSS, SST, SSH, *θ*), we would need to incorporate any and all uncertainties that exist in the inputs, observations, model parameters, and model framework and be able to sample from them effectively. Due to the high‐dimensionality of the problem, this is computationally infeasible and therefore we must rely on adequate approximations. Appendix A details the multiple probabilistic machine learning techniques considered in our experiments: dropout, covariance parameterization, sampling‐based models, and deep ensembles.

## 4 Experimental Results

Table 1 provides a summary of 10 different methods that we tested on each experiment (for brevity, not all results are presented), along with an approximate number of parameters necessary for each model, divided by the number of output grid points, and the corresponding method of MLD variance estimation. The “Linear” and “VLinear” methods are implementations of Equation 5, but in the “VLinear” model the variance is also estimated by a “Linear” model. The majority of algorithms are based on the convolutional neural network architecture (CNN). For a schematic of the models implemented in this text, see the appendix. The CNN is widely used in image processing problems because it couples inputs spatially by use of kernels, instead of matrix multiplication used in dense neural networks. The three generic variation estimation methods tested are: (a) Parameterization, either in the form of a linear parameterization, where the variance is an affine function of SSS, SST, and SSH, or a CNN parameterization, where the variance is produced as an additional filter of the CNN output along with the MLD estimate; (b) Direct Sampling, where a single model must be run multiple times to produce a sample‐based estimate of the variance; and (c) Ensemble sampling, where multiple models are trained, as in the Deep Ensemble technique (Lakshminarayanan et al., 2017), and the variance is estimated from a random sampling of outputs from the collection of models. We tested many more permutations and combinations of these models and variance estimation methods but only present the highest performing models for publication. The ResNet and Deep CNN models are specific structures of CNN's that couple data throughout different layers of the network, so as to avoid the problem of vanishing gradients (He et al., 2016).

**Table 1:** Summary of Implemented Models

Model	∼# of params. per grid point	Variance est. method
Linear	4	N/A
VLinear	8	Linear parameterization
ANN	2,178	N/A
CNN	69	Ensemble sampling^a^
VLCNN	73	Linear parameterization
VCNN	69	CNN parameterization
Dropout	69	Direct sampling
CVAE	334	Direct sampling
ResNet	102	Ensemble sampling^a^
Deep (V)CNN	7,020	CNN parameterization

*Note*. See Appendix A for a summary of each variance estimation method and Appendix B for a more information about the structure of each machine learning architecture.

^a^ For ensemble‐based estimation of variance, the total number of parameters is multiplied by the number of ensemble members.

In the CESM POP2 Ocean Model experiments, the machine learning model estimates are compared directly against ocean model MLD (taken as “truth”) at each grid cell. In the Argo data set experiments, the machine learning models are compared in observation space, at the Argo profile locations (using interpolation, Gaussian process regression). In the Argo experiments we also compare the machine learning approaches to kriging, in order to compare the results to a method that only utilizes the Argo profiles themselves. Specifically, we implement an Ordinary Kriging scheme, which we call “OI” for optimal interpolation, with a (spatial) spherical kernel chosen via cross‐validation and parameters optimized via maximum likelihood. The OI approach only uses the in situ MLD standard anomaly observations, with no sea surface information, to make gridded estimates. Therefore, even during the out‐of‐sample prediction experiments, the OI's error statistics for a given week are calculated using only that week's data. The OI is trained only on half of the available data for a particular week and then is asked to estimate the MLD at the withheld locations.

We use a variety of metrics in our testing, categorized into deterministic and probabilistic metrics. For deterministic metrics, we use the (relative) mean absolute error (MAE) and Pearson correlation coefficient. We use the typical definition of mean absolute error,

(9) $$\text{Relative}\;\text{MAE}=\frac{1}{n}\sum_{i=1}^{n}\frac{\left|{\left(d_{o}\right)}_{i}-L{\left(d\right)}_{i}\right|}{\left|{\left(d_{o}\right)}_{i}\right|}.$$

where we average over *n*, the number of observation available at a given week. A lower MAE is more accurate.

MAE is a convenient metric in that it captures the mean prediction error, but it does not describe the relationship between the predictions and observations and it fails to capture information about the uncertainty of the predictions. To compensate for the first deficiency, we rely on the Pearson correlation coefficient (correlation) to provide insight into the existence of (linear) relationships between predictions and the data. For reference, correlation is defined as

(10) $$\text{Correlation}=\frac{\sum_{i=1}^{n}(L{\left(d\right)}_{i}-\bar{L\left(d\right)})({\left(d_{o}\right)}_{i}-\bar{d_{o}})}{\sqrt{\sum_{i=1}^{n}(L{\left(d\right)}_{i}-\bar{L\left(d\right)})}\sqrt{\sum_{i=1}^{n}({\left(d_{o}\right)}_{i}-\bar{d_{o}})}}$$

where the $\bar{\text{overline}}$ symbol represents the sample mean operation. A correlation coefficient closer to 1 is considered more skillful.

In addition to the deterministic metrics, we use the (relative) Continuous Ranked Probability Score (CRPS), to provide a probabilistic error metric analogous to MAE, and we analyze the error distributions using the Kullback–Leibler divergence (KL Divergence or *D*_KL_) and Kolmogorov–Smirnov (KS) statistic. These metrics are useful in determining the distance between the predicted and observed probability distributions and measuring the calibration of each model.

For forecast distributions with a finite second moment, the relative CRPS can be defined as

(11) $$\text{Relative}\;\text{CRPS}=\frac{1}{n}\sum_{i=1}^{n}\left(\sum_{j=1}^{n_{e}}\frac{|{\left(d_{o}\right)}_{i}-L{\left(d\right)}_{i}^{j}|}{n_{e}|{\left(d_{o}\right)}_{i}|}-\sum_{j=1}^{n_{e}}\sum_{k=1}^{n_{e}}\frac{|L{\left(d\right)}_{i}^{j}-L{\left(d\right)}_{i}^{k}|}{2n_{e}^{2}|{\left(d_{o}\right)}_{i}|}\right)$$

where *n*_*e*_ represents the number of forecast estimates available per observation. The CRPS collapses to the MAE when the measurement is deterministic (*n*_*e*_ = 1), but accounts for ensemble spread in probabilistic forecasts when *n*_*e*_ > 1. The CRPS is the probabilistic equivalent of the Relative MAE.

We estimate the discrepancy between the forecast and observed distributions using the KL Divergence and KS statistic. Both metrics make use of the error distribution, *p*(*x*), defined as

(12) $$p\left(x\right)=P\left[\frac{{\left(d_{o}\right)}_{i}-\bar{L{\left(d\right)}_{i}}}{s_{i}}=x\right],$$

where *s*_*i*_ is the estimate of the standard deviation for *L*(*d*)_*i*_ and *P*[*t*] is the probability of the event *t*. We estimate *p*(*x*) through the use of a histogram. Theoretically, a “well‐calibrated” model produces an error distribution that is approximately normal, that is, *p*(*x*) should be approximately equal to $q\left(x\right)=e^{-x^{2}/2}/\sqrt{2\pi }$. We calculate the KL Divergence and KS statistic to measure the discrepancy between the forecast error distribution and the theoretical error distribution. The KL Divergence is defined as

(13) $$D_{KL}\left(p,q\right)=\int_{-\infty }^{\infty }p\left(x\right)\log\frac{p\left(x\right)}{q\left(x\right)}dx.$$

The KL Divergence heavily weights errors in the tail of the theoretical distribution *q*, that is, outliers. We also consider the KS goodness of fit statistic because it is more sensitive to errors near the mode of *q*, rather than in the tails of *q*. The KS statistic is a norm on the cumulative distribution functions of *p* and *q*,

(14) $$\text{KS}=\sup_{x}\left|\int_{-\infty }^{x}p\left(x\right)-q\left(x\right)dx\right|.$$

We calculate these statistics by estimating a histogram of *p*(*x*) and *q*(*x*) and performing discrete integrals and maxima. A lower value of these error distribution metrics indicates a better calibrated forecast distribution.

### 4.1 CESM POP2 Ocean Model Data Results

Conclusions from the Argo experimental results are problematic on their own because of observational uncertainties and the limited availability of data to provide verification (50 out of 200 weeks are afforded for testing and verification). The CESM POP2 Ocean Model experiments are an idealized test environment to provide answers to three questions in a relatively data rich environment (when compared to the Argo data set) without observational uncertainty. First, to what extent is there a relationship between the sea surface input data (SSS, SST, SST) and MLD in the two regions being studied (and how does it depend on the region)? Second, what is the relative performance between machine learning models and, furthermore, which models perform “best”? Third, how do each of the input variables affect our machine learning model MLD predictions? The following ocean model results are calculated from the validation data set, see Section 2 for details. We hypothesize that the answers to these questions derived from the ocean model experiments are approximately transferable to the Argo experiments. Training the machine learning models on the ocean model data set also allows us to use transfer learning by reusing the parameters as starting points for learning in the Argo experiments.

#### 4.1.1 Deterministic Metrics

**Figure 4:**
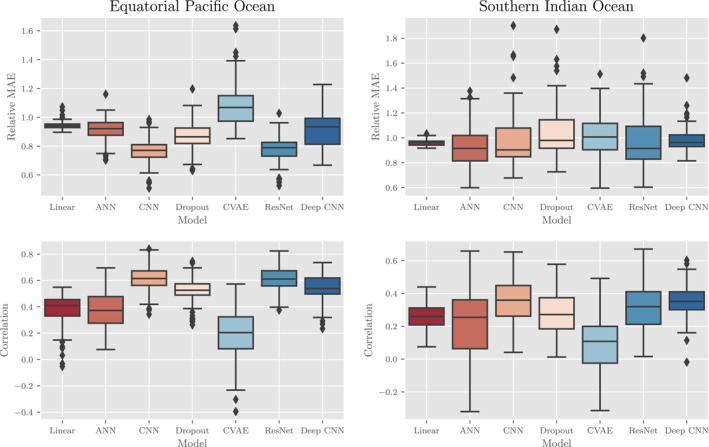
Deterministic results of various machine learning methodologies as applied to the CESM POP2 Ocean Model data experiment. Boxes capture 25%–75% of the weekly errors with the middle line representing the median error. Dots are considered outliers—values which are 1.5 × lower/upper quantile. In the top row, a comparison of the relative mean absolute errors (MAEs) for each method in the (left) equatorial Pacific Ocean (EPO) and (right) southern Indian Ocean (SIO). In the bottom row, a comparison of the correlation coefficients between predictions and truth in the (left) EPO and (right) SIO. Each metric is unit‐less and a lower MAE and higher correlation are equated with better performance. For each pair of graphs, notice the different scales for the EPO and SIO results as performance is generally better in the EPO compared to the SIO.

Our findings indicate that there is a moderately predictive relationship between the sea surface variables and MLD in the ocean model data in the equatorial Pacific (EPO) and, to a lesser extent, the southern Indian (SIO) ocean as tested on the validation data set (roughly all 5‐day weeks in 1998). The deterministic results in Figure 4 provide the (top) Relative MAE (as a %) and (bottom) correlation coefficient for a variety of the models tested in both the (left) EPO and (right) SIO. From a broad perspective, a correlation coefficient of 0.6, on average, between the CNN outputs and validation data in the EPO indicates that there exists a moderate relationship between sea surface variables and MLD (all values presented in the plots are statistically significant). On the other hand, relative absolute errors for the CNN outputs in the EPO are, on average, 80%. These two facts together would indicate that while the CNN can reasonably learn spatial patterns and relative magnitudes of grid points from the data, there exists significant error. By both metrics, most of the models are worse in the SIO, where correlations top out at about 0.4 and the MAEs range between about 0.9 and 1.0. We hypothesize that the stronger interannual variability in the EPO induces a stronger intrinsic physical relationship between the the sea surface and the mixed layer anomalies, which explains the relatively better performance in the EPO than in the SIO across every model. It remains unclear, however, whether this effect is truly causal. Finally, these deterministic results also indicate that more expressive machine learning models, such as the ANN, ResNet, and Deep CNN, do not necessarily have better predictive power. However, this last finding may be an artifact of the still relatively small data set used for training (in comparison to large computer‐vision data sets that these models are typically trained on) and the impact on overfitting.

**Figure 5:**
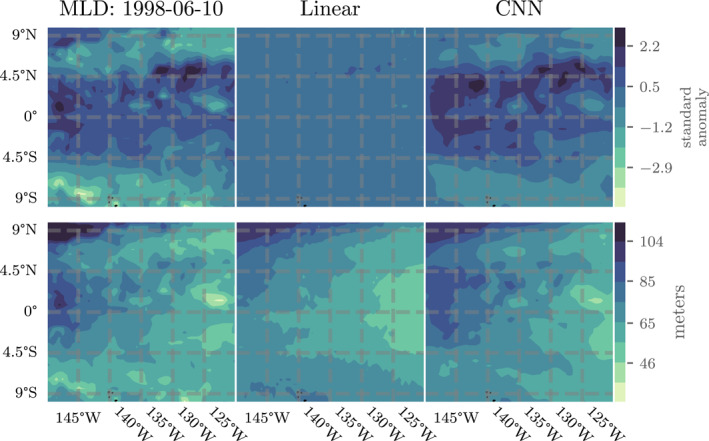
Filled contour plots showing (left) mixed layer depth, (middle) Linear, and (right) Convolutional Neural Networks (CNN) model predicted outputs for the equatorial Pacific Ocean (EPO) for the 5‐day week starting on June 10, 1998. The top row shows MLD standard anomalies while the bottom row shows the corresponding MLD (in meters) with climatologies reintroduced. As the images suggest, the pattern correlation with the true MLD is much higher for the CNN (Pearson's *r* = 0.83) than the Linear model (*r* = 0.37).

**Figure 6:**
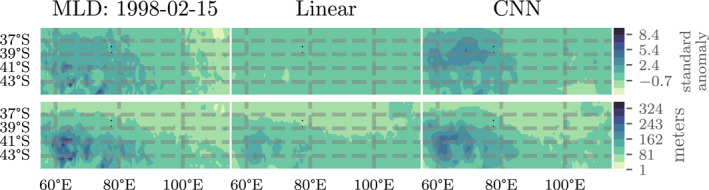
Details as in Figure 5 but for the southern Indian Ocean (SIO) for the 5‐day week starting on February 15, 1998. As in Figure 5, the pattern correlation with the true MLD is much higher for the CNN (*r* = 0.65) than the Linear model (*r* = 0.25).

It is seemingly significant that the Linear model provides results seemingly competitive with the other machine learning models (possibly indicating that the majority of the relationship between input data and MLD is linear), but these statistics can also be somewhat misleading. A visual comparison of the model outputs sheds additional light on the quality of MLD predictions. Figures 5 and 6 show maps of MLD and predictions from the Linear and CNN models for the best performing weeks (for CNN) from the EPO and SIO, respectively. In the top row of each figure we show the MLD standard anomalies (which are the direct output from each model) and in the bottom row we show the corresponding MLD (in meters, with climatologies). While Figure 4 suggests that the Linear model provides reasonably predictive outputs, the visual maps indicate that its outputs actually have very small amplitudes and cannot replicate spatial variations on the same scale as the true MLD standard anomalies. The CNN outputs, however, provide spatial variations that look closer to reality but still miss small‐scale details. These qualitative results are reflected to some degree in higher correlations between MLD and CNN predictions than between MLD and Linear predictions: *r* = 0.83 and 0.65 for CNN, and *r* = 0.37 and 0.25 for Linear in the EPO and SIO, respectively. However, least squares linear regression (with intercept) of MLD on the CNN or Linear model predictions reveal steeper slopes in the Linear model than the CNN: 2.5 compared to 1.2 in the EPO and 1.5 compared to 0.7 in the SIO. That is, the Linear model is not even fitting the data as well as the correlation coefficient indicates in these examples. In combination with the deterministic results, these figures indicate that relative error and correlation coefficient do not necessarily capture all of the behavior we might want from a MLD model or the relative strengths of the CNN‐based machine learning models, which represent spatial structures well but do not always capture their location and extent.

#### 4.1.2 Probabilistic Metrics

**Figure 7:**
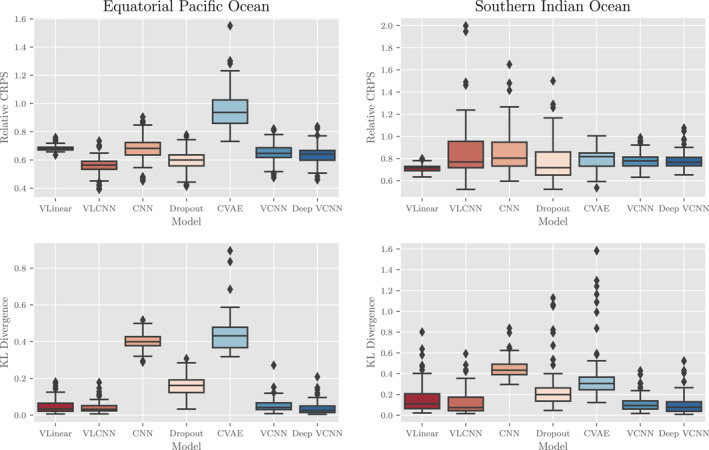
Probabilistic results of various probabilistic machine learning techniques and architectures as applied to the CESM POP2 Ocean Model data experiment. In the top row, a comparison of the relative continuous ranked probabilistic score (CRPS) for each method in the (left) equatorial Pacific Ocean (EPO) and (right) southern Indian Ocean (SIO). In the bottom row, a comparison of the KL Divergence between the normalized errors and a standard normal distribution in the (left) EPO and (right) SIO. Each metric is unit‐less and lower scores equate to better performance. For each pair of graphs, notice the different scales for the EPO and SIO results as performance is generally better (with less extreme outliers) in the EPO compared to the SIO.

Our results also indicate that parametric variance methods outperform other sampling‐based techniques for estimating MLD uncertainty in the CESM experiments. The probabilistic results in Figure 7 show the relative CRPS and KL Divergence metrics for the various probabilistic machine learning models we tested. The parametric variance models, for example, “VLinear,” “VLCNN,” “VCNN,” and “Deep VCNN,” outperform the sampling‐based strategies, for example, “CNN” (deep ensemble), “Dropout,” and “CVAE,” especially in terms of the KL Divergence (calculated on the error distribution). The KL Divergence is particularly sensitive to outliers, indicating that the sampling‐based techniques underestimate uncertainty in MLD standard anomaly more often than the parametric techniques. This should not be surprising since the parametric techniques can include variance inflating terms (via a Bayesian prior probability distribution placed on the variance parameters) in the optimization process while the sampling techniques (especially the deep ensemble techniques) rely on variation in the input data alone.

**Figure 8:**
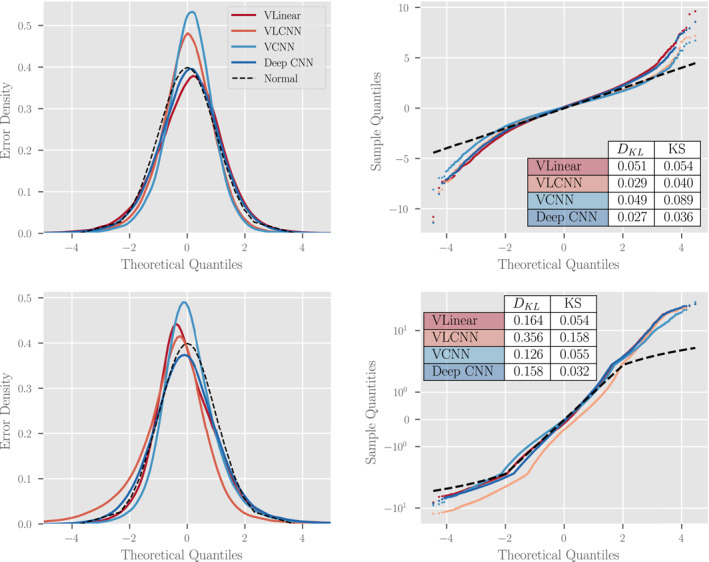
A study of the distribution of normalized errors for four of the top performing probabilistic methodologies and architectures from Figure 7 assessed by grid‐point. Each model assumes that the difference between model predictions and reality, normalized by the model standard deviation, should be approximately normal with mean zero and unit variance. For (top) EPO and (bottom) SIO, we present (left) a visual comparison of the error distribution probabilistic density functions and (right) a quantile‐quantile comparison of the given error distribution and a standard normal distribution (dotted black line, optimal) with a table showing the KL Divergences and relative *L*_2_ norm between sample and theoretical distributions (see text for definitions). Notice the semi‐log scale on the bottom‐right plot. For an interpretation of the right plots, see the accompanying discussion in the text.

The probabilistic results indicate that some of the probabilistic machine learning algorithms are reasonably well‐calibrated. Figure 8 shows the (left) error distributions for the top four best‐performing machine learning algorithms from Figure 7 and the reference standard normal distribution as well as (right) quantile‐quantile (QQ) plots demonstrating the deviation from normality for each distribution along with KL Divergence and KS goodness of fit metrics. From the QQ plots we find that the error distributions are well‐calibrated for the data within two standard deviations of the mean (on the interval [ − 2, 2]). Outside of that interval, the deviation between error and reference distribution indicates that the models' error distributions have fatter tails than expected—the models significantly underestimate uncertainty slightly more often than expected. We measure the discrepancy between each error distribution and the reference distribution through two metrics: the KL Divergence and KS statistic. The KL Divergence places more weight on errors incurred at the tail of the reference distribution while the KS statistic places more weight near the mean of the reference distribution. From these metrics, we find that the the linearly parameterized models (“VLinear” and “VLCNN”) fit the mean of the distribution well but have poorer performance in the tails. In total, the “Deep VCNN” model has the best overall model calibration of all models tested on the CESM POP2 Ocean Model data and visual inspection of the error distribution indicates good predictability, although at an increased computational cost (see Table 1).

#### 4.1.3 Model Sensitivities

**Figure 9:**
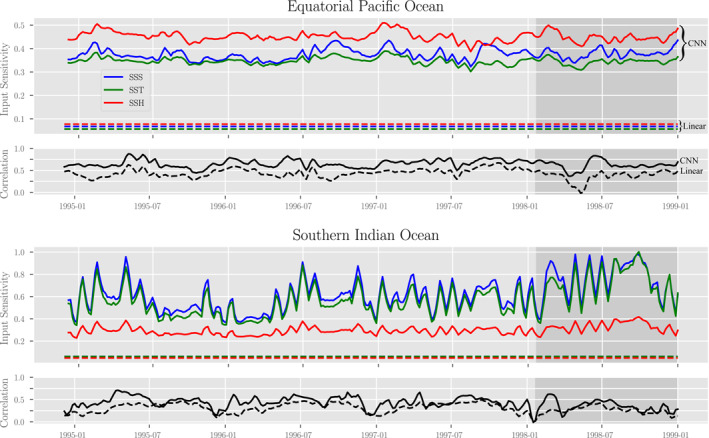
Model sensitivities to each of the three input variables SSS, SST, and SSH as measured by the method of Integrated Gradients (see the text for definition) and correlation of model outputs with truth in the (top half) EPO and (bottom half) SIO. Solid lines correspond to sensitivities and metrics with respect to the CNN model while dashed lines represent sensitivities and metrics with respect to the linear model. The units of the sensitivities share the unit and scale of the model outputs, which are the (unit‐less) MLD standard anomalies. The shaded region signifies the validation data.

As a means to understand the behavior of the machine learning models, we explore the sensitivity of the MLD outputs with respect to the input sea surface variables via a technique known as “Integrated Gradients” (Sundararajan et al., 2017). This technique computes a line integral of model derivatives to compute a total sensitivity. Specifically, we calculated this quantity, which we refer to as “Input Sensitivity,”

(15) $$InputSensitivity\left(x,x_{0}\right)=\left(x-x_{0}\right)\int_{0}^{1}\frac{df}{dx_{i}}(x_{0}+\alpha \left(x-x_{0}\right);\theta )d\alpha ,$$

where *f* is the MLD model, x=(SSS,SST,SSH), and *x*_0_ is some baseline initial condition for the line integral. This technique is sensitive to initial baseline and many choices are available depending on the context (a common baseline for images is the zero initial condition). For our study, we compute the integrated gradient corresponding to 50 random draws from a Gaussian distribution with *σ* ^2^ = 2 and take an ensemble average. The results from this computation are shown in Figure 9, where we compare the sensitivities of the CNN and Linear models.

From our sensitivity studies we find that the CNN model is much more sensitive to the input variations than the Linear model, which may explain the disparate differences in output maps (Figures 5 and 6), while the relative ordering in sensitivities between the three input variables is the same for each model. In the EPO, the models are more sensitive to changes in SSH, followed by SSS and SST. In the SIO this relationship is somewhat flipped, with models being more sensitive to changes in SSS and SST than SSH (and more sensitive in general). This difference in relative orders might suggest qualities about the dynamic processes giving rise to MLD in these respective regions, for example, vertical advection and thermocline displacement, horizontal advection, or surface processes. The orderings may also reflect differences in the relative magnitude of subseasonal variability compared to the seasonal cycle (which is small in the EPO and large in the SIO). It is particularly intriguing and somewhat surprising that salinity has a higher input sensitivity than temperature in both regions, and it is tempting to speculate on the physical basis of these results. Perhaps the sharp background meridional salinity gradient highlights horizontally advective MLD anomalies better than the temperature structure, which is more strongly coupled to the atmosphere. Conversely, perhaps the impacts of vertically variable (i.e., sheared) horizontal advection of salinity more directly influences aseasonal MLD variability than temperature via the upper‐ocean stratification budget (there is evidence for this effect during winter in the Southern Ocean [DuVivier et al., 2018; Small et al., 2020]). However, we find it difficult to draw conclusions about the underlying physics from the input sensitivities without a more detailed investigation, which is left for future work. In addition, more tests will be needed since the difference in results could also be an artifact of the model's ability to actually learn a physical relationship (which was less significant in the SIO). There is only a mild (0.2 in the EPO) to no (0.05 in the SIO) correlation between the error metrics and these sensitivities, however.

Finally, it should be noted that the behavior of the sensitivities and correlation statistics (shown in Figure 9) as well as error statistics (not shown) do not seem to have a visual dependence on time (across the training, test and validation data sets). Furthermore, the temporal behavior of the machine learning model performance and linear model performance track closely. These facts suggest that there is minimal overfitting from the machine learning models.

However, there are a few subtle patterns in the correlation time series of possible scientific interest (but without obvious analog patterns in the input sensitivities). First, correlations between CNN predictions and MLD are higher in Boreal Summer/Austral winter and lower in Boreal winter/Austral summer at both sites. This seasonal pattern in predictability is clearest in the SIO, where correlations between both model predictions and the MLD reach a consistent minimum in the Austral summer/Boreal winter, dropping below 0.25 near the new year in all 5 years when the climatological MLD is shallow but was recently deep (Figure 2). The Austral wintertime peaks in predictability in the SIO are less clear, but still qualitatively apparent. In the EPO, the CNN model correlations with MLD notably exceed 0.75 in all 4 years during the early Boreal summer/Austral winter, when the MLD seasonal climatology is relatively shallow. However, it is not clear that correlations in the EPO exhibit such a pattern in the Linear model. In any case, the physical basis of these seasonal variations in predictability of MLD from surface variables are unclear but of interest for future work.

### 4.2 Argo Float Data Results

The results from the CESM POP2 Ocean Model data suggest that there exists a moderately strong relationship between sea surface variables and the MLD in the EPO (*r* ≈ 0.6), and to a lesser extent in the SIO (*r* ≈ 0.4). Using the parameters learned in the previous experiment as initial conditions, we take the leading machine learning models—the linear model, “VLinear,” and three CNN models, “VLCNN,” “VCNN,” and “Deep CNN”—and retrain to fit the optimally interpolated satellite sea surface data and Argo MLD profile data set. As opposed to the ocean model data, there are additional sources of model and reconstruction error that influence and complicate the ability for the machine learning models to learn a corresponding MLD relationship. Each of the inputs is subject to different reconstruction errors and biases while the calculation of the gridded climatology for the Argo data sets introduces further biases. Furthermore, the sparsity of the Argo data adds an additional difficulty when training data‐driven models, especially deep learning models with many parameters.

Ultimately, the machine learning models that we train will be compared against the results of applying optimal interpolation (kriging) to the Argo data alone to produce smooth MLD spatial maps (we label these results as OI). While OI is known to be a sub‐optimal process for obtaining gridded estimates of the MLD, as data assimilation methods that combine multiple data sources and ocean models are usually more accurate, the use of OI in our study is to compare strictly data‐driven methodologies and to have a direct comparison with a method that only has access to the Argo observations themselves to learn spatial variability and uncertainty. To estimate the corresponding error metrics for this methodology we randomly sample half of the available Argo profiles for a given week, 10 separate times, fit the OI and produce the corresponding map, and calculate a variety of metrics using the left out data (averaged over the 10 samples). It is important to remember that, in contrast to the machine learning models, the OI methodology has direct access to the MLD values and the errors represent spatial out‐of‐sample errors. These errors are not necessarily equivalent to the errors for the machine learning model, but serve as an important benchmark for potential practitioners.

#### 4.2.1 Deterministic Metrics

**Figure 10:**
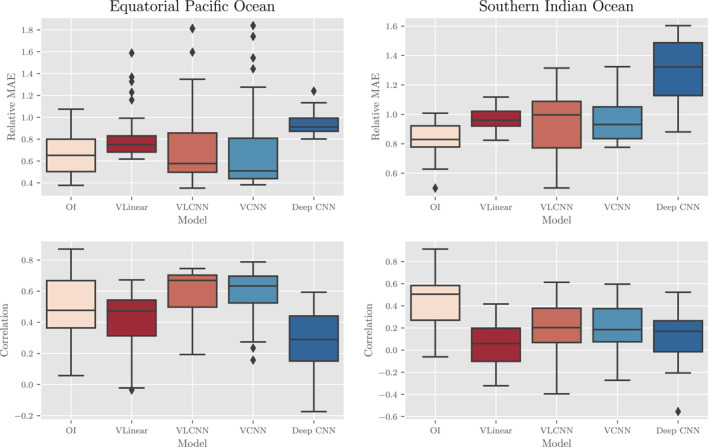
Deterministic results of various machine learning methodologies and OI as applied to the Argo profile data experiment. Boxes capture 25%–75% of the weekly errors with the middle line representing the median error. Dots are considered outliers ‐ values which are 1.5 × lower/upper quantile. In the top row, a comparison of the relative mean absolute errors (MAEs) for each method in the (left) equatorial Pacific Ocean (EPO) and (right) southern Indian Ocean (SIO). In the bottom row, a comparison of the correlation coefficients between predictions and truth in the (left) EPO and (right) SIO. Each metric is unit‐less and a lower MAE and higher correlation are equated with better performance. For each pair of graphs, notice the different scales for the EPO and SIO results as performance is generally better in the EPO compared to the SIO.

Deterministic results for the Argo data experiment suggest that the machine learning models can produce MLD maps competitive with OI, as measured by relative MAE and correlation, especially in the EPO. Figure 10 shows the (top) Relative MAE and (bottom) correlation coefficient for each model that we tested in the (left) EPO and (right) SIO. In the EPO for example, OI and machine learning algorithms have comparable error metrics with the “VLCNN” and “VCNN” algorithms generally having highest median correlation coefficients and lowest median relative MAEs. Conversely, the “Deep CNN” algorithm has worse metrics than OI or the other algorithms. With that caveat that the performance of the machine learning models is worse absolutely and relative to OI in the SIO, the information that the machine learning models appear to have learned to extract from the surface variables in the EPO is as informative, per these metrics, as nearby MLD Argo observations themselves in estimating MLD spatiotemporal variability (for maps of typical Argo profile distributions, see Figures 12 and 13 that are discussed further in a following paragraph). The relatively poor performance of OI by these metrics is a reflection of the weak spatial correlation over the large distances between sparse neighboring Argo profiles, as well as a reflection of the value of dense surface data.

The comparison between the EPO and SIO reiterates the important result derived from the ocean model that the relationships between MLD and surface variables differ regionally. The performance metrics are worse on all algorithms in the SIO compared to the EPO. Although the ordering of the machine learning algorithms by MAE and correlation is similar between the SIO and EPO, the OI has a clear performance advantage, vis‐a‐vis the machine learning algorithms, in the SIO, having smaller median relative MAE and higher median correlation (Figure 10). It appears, then, that the ability for the machine learning algorithms to extract a relationship between the surface and subsurface is weaker in the SIO than in the EPO. At a minimum, this difference in the EPO and SIO performances suggests a fundamental difference in the relationship between surface and MLD variability that is due to the aseasonal dynamics of the region of interest.

Finally, the deterministic results of the Argo data set appear to be analogous to CESM POP2 data set errors in the EPO but noticeably worse in the SIO. While comparisons between Figures 4 and 10 should be taken lightly because of methodological and modeling differences, we find that the relative MAEs and correlations in the EPO are similar, with small statistical differences between “CNN” CESM POP2 performance and “VLCNN” or “VCNN” Argo performance. A comparison among correlations in the SIO, however, reveal noticeably worse performance across machine learning models, particularly from the “Deep CNN” model. This relative performance may indicate that the relationship among SSS, SST, and SSH in the SIO is weaker or harder to identify via machine learning. That is, the degradation of model performance in the SIO may indicate that the machine learning algorithms require additional data to estimate a relationship comparable in strength to the SIO results from CESM POP2 in Figure 4. It may also be the case that a relationship between the surface and subsurface aseasonal variability in the SIO of the same strength as in the CESM POP2 ocean model does not exist in the real ocean or cannot be learned by this machine learning methodology.

#### 4.2.2 Probabilistic Metrics

**Figure 11:**
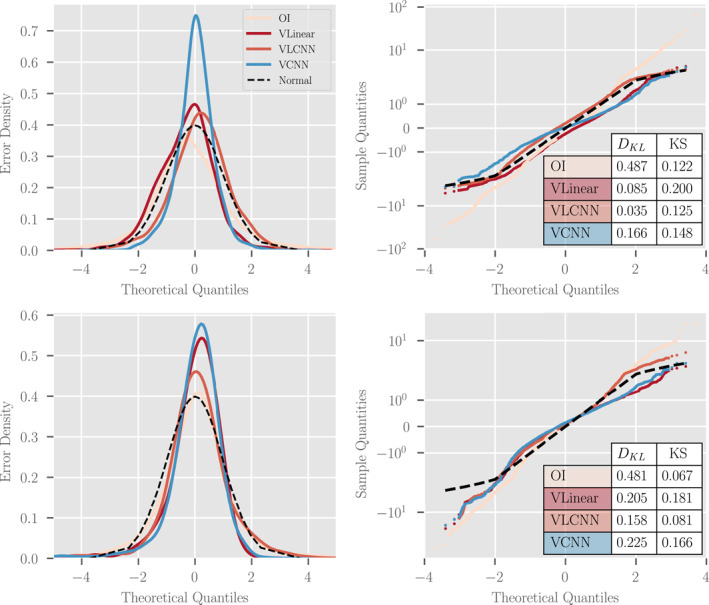
Probabilistic calibration for temporal out‐of‐sample prediction as in Figure 10. Statistics are calculated on each datapoint for the 50 withheld validation weeks. For (top) EPO and (bottom) SIO, we present (left) a visual comparison of the error distribution probabilistic density functions and (right) a quantile‐quantile comparison of the given error distribution and a standard normal distribution (dotted black line, optimal) with a table showing the KL Divergences and relative *L*_2_ norm between sample and theoretical distributions (see text for definitions). Notice the semi‐log scale on the right plots.

Calibration results for these models and the Argo data set show reasonably good predictability, even in the SIO. Error distribution histograms and quantile‐quantile (QQ) plots for this experiment are shown in Figure 11 with (top) EPO and (bottom) SIO. In the EPO, all distributions, except the “VCNN” distribution, show a reasonable resemblance to a standard normal. More of the machine learning model distributions exhibit qualitatively significant deviations from a standard normal in the SIO than the EPO, but the OI distribution looks more like the normal distribution in the SIO than in the EPO. The QQ plots highlight that the tails of the distribution are quite like a standard normal distribution in the ML models of the EPO (including “VCNN”), but the tails of the distribution are too‐heavy in the OI. Likewise, the OI has too‐heavy tails in the QQ plot from the SIO, and the ML‐modeled tails differs more strongly from the standard normal in the SIO than the EPO. The heavy‐tail behavior is summarized in the KL Divergence, which is heavily influenced by deviation from normality in the tails of the distribution and takes highest values for the OI. Conversely, the KS statistic is less impacted by outliers and takes lowest values for the OI. The performance of the ML models are more consistent between the two metrics and regions in that “VLCNN” takes the lowest values of both the KL divergence and the KS statistic in both regions. It should be noted, however, that the variance in the OI model and the variance from the machine learning models are different. The uncertainty in OI predictions are due, mostly, to spatial effects, that is, the model is more uncertain in locations that are far away from observations, whereas the machine learning models have learned dynamic variance from the sea surface data. Still, the out‐of‐sample relationships that the machine learning models (specifically, the “VLCNN”) have learned from the Argo data is competitive with OI.

**Figure 12:**
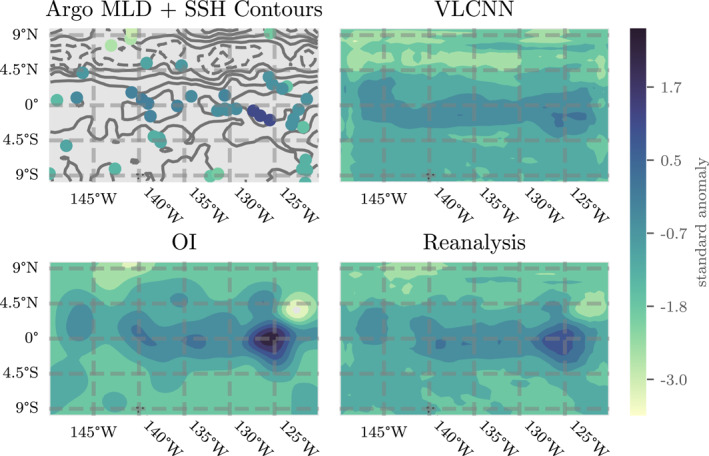
A visual comparison of Argo profiles and estimated contour maps. From left to right, scatter plot of Argo profile locations with filled mixed layer depth (MLD) values overlayed on gray SSH contours with 0.05 m contour spacings, filled contour plots of OI estimated MLD, VLCNN estimated MLD, and reanalysis estimated MLD (see text for definition) for the equatorial Pacific Ocean (EPO) for the 7‐day week starting on April 24, 2015. The top row shows MLD standard anomalies while the bottom row shows the corresponding MLD (in meters) with climatologies reintroduced.

**Figure 13:**
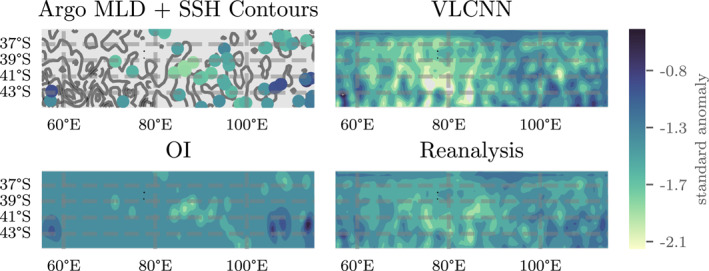
A visual comparison of Argo profiles and estimated contour maps. Counterclock‐wise from upper‐left, scatter plot of Argo profile locations with filled mixed layer depth (MLD) values overlaid H contours with 0.15 contours lines, filled contour plots of OI estimated MLD, VLCNN estimated MLD, and reanalysis estimated MLD (see text for definition) for the southern Indian Ocean (EPO) for the 7‐day week starting on April 24, 2015. The top row shows MLD standard anomalies while the bottom row shows the corresponding MLD (in meters) with climatologies reintroduced.

The visual, qualitative features of the machine learning MLD maps appear promising as they capture features smaller in scale than in typical OI maps and many of the features that appear in the inputs. Figures 12 and 13 show Argo profile locations and MLD values overlaid on SSH contours, along with OI, VLCNN, and Reanalysis MLD estimated maps for April 24, 2015 in the EPO and January 16, 2015 in the SIO, respectively. These dates are arbitrarily taken from the validation data set with the correlation coefficient of the machine learning maps in each plot being approximately 0.6. The OI maps are estimated directly from the Argo profile locations and MLD values, while the VLCNN maps are estimated from sea surface inputs. Because the VLCNN maps do not have access to the Argo MLD data, we can define a reanalysis that updates the machine learning maps from the available data,

(16) $$\begin{array}{ccc} \hat{d} & = & \underset{d}{\text{arg min}}-\ln p\left(d|d_{o},d_{m}\right),\\ & & =\underset{d}{\text{arg min}}{\left(d-d_{m}\right)}^{T}\Sigma^{-1}\left(d-d_{m}\right)+{\left(Ld-d_{o}\right)}^{T}V^{-1}\left(Ld-d_{o}\right), \end{array}$$

where *d*_*m*_ is the machine learning MLD estimate, *d*_*o*_ is the Argo observations, Σ is the machine learning covariance estimate, and *L*, *V* are the Gaussian Process mapping and covariance matrices.

In both the EPO and SIO, each map provides an estimated map that roughly agrees with the Argo samples. The spatial effect of the SSH contours is evident in the machine learning outputs, and the fact that many of these features correlate with details in the Argo MLDs means that the machine learning models were able to learn these smaller scale relationships that hold out‐of‐sample from the training data. The OI, in comparison, does not visually capture small‐scale features apparent in the input data and presumed to exist in the MLD (at least from maps in Figures 5 and 6). In the SIO, in particular, the fact that the OI has a slightly higher correlation coefficient than the VLCNN map reminds readers that the error statistics do not provide a complete encapsulation of the quality of MLD reconstruction from each method. Clearly there is some qualitative benefits from the machine learning maps that are not entirely captured by the deterministic or probabilistic errors.

## 5 Conclusion and Discussion

The ocean mixed layer interacts with the atmosphere and deep ocean on a multitude of spatial and temporal scales. Heat exchange between these bodies has significant impacts on the dynamics of subseasonal and interannual (aseasonal) variability and can influence the behavior of dominant modes of variability (i.e., ENSO, MJO, and tropical cyclones). The proliferation of Argo floats has dramatically increased the number of observations of the ocean over the last two decades but Argo profiles are still too sparse to resolve fine spatiotemporal features of the MLD that are apparent in state‐of‐the‐art global ocean models. Satellite data, however, is able to provide fine resolution gridded maps of sea surface variables, but cannot observe subsurface.

The first goal of this work was to analyze the extent to which satellite observations of sea surface variables can provide information useful for estimating the MLD. We built several machine learning models to learn such a relationship based on available data. In order to test this methodology on a self‐consistent system, while minimizing reconstruction and interpolation errors, we first trained machine learning models on CESM POP2 ocean model output. On this ocean model output we found that the relationships learned by the data‐driven models had a moderate (in the SIO, *r* ≈ 0.4) to strong (in the EPO, *r* ≈ 0.6) correlation with MLD on the temporally out‐of‐sample data. Furthermore, we found that several of the machine learning models exhibit good predictability and calibration (Figure 8). The correlations and error distributions do not exhibit much temporal dependence, indicating that there is minimal overfitting (Figure 9).

Following this experiment, we tested our methodology on the Argo data set. The number of weeks available for testing and validation data is not wholly sufficient to form conclusions on the source of MLD variability in this data set. However, we found that in terms of both deterministic (Figure 10) probabilistic metrics (Figure 11), and visual qualitative behavior (Figures 12 and 13), the machine learning model results suggest that the satellite data is equally if not more useful in estimating spatiotemporal variability in MLD values and uncertainties than MLD observations alone, given that sufficient MLD observations are available for out of sample training. The relative performance between these methods can depend on the location of interest and the characteristics of the variability (e.g., between the Southern Indian Ocean and Equatorial Pacific Ocean), but we believe that the machine learning methodology can be widely applicable and competitive with optimal interpolation approaches globally in the future. Therefore, including surface information together with in situ MLD estimates may be useful for generating improved reanalyses of the upper ocean under these circumstances. The comparison with OI is meant as a comparison with an observation‐based methodology that only has access to Argo profile observations that can yield a comparison of the relative value of surface information. The comparison is not meant as a statement on of the value of OI or a statement that OI is state‐of‐the‐art, which it is not (especially in comparison to multivariate reanalyses and data assimilation products).

The second goal of this work was to use sophisticated probabilistic learning approaches to better understand the probability distribution of the MLD. The primary modeling assumption in our machine learning methodology was the normality of the distribution of the errors between the model estimates and observations (Equation 1). Calibration results in both Figures 8 and 11 suggest that this modeling assumption is reasonable and that the machine learning algorithms produce reasonably well‐calibrated MLD estimates. There is, however, a not‐insignificant number of outliers across error distributions that might be improved given additional data. We also found that parameterized distributions outperform sampling‐ and ensemble‐based uncertainty quantification techniques. This suggests that Gaussian parameterizations of the conditional uncertainty in MLD spatiotemporal variability is sufficient, but sampling techniques might also be improved in the future with additional data.

This work is an initial step into machine learning modeling of the MLD and there are several avenues for continued methodological and oceanographic research. First, the results in this study are regional test cases chosen to reveal how the variability of the MLD impacts the ability of the machine learning methods to learn a functional relationship between the surface variables and the MLD. The machine learning models trained here, specific to each region, cannot immediately be applied to a different region, since the dynamics learned change from region to region. Future work will expand this regional approach to a global scale, but will necessitate additional computational resources. Second, further research and data is needed to derive better estimates of the conditional posterior probability distribution of the MLD. This research could include weight uncertainty, more sophisticated sampling strategies, covariance regularization, or other neural network architectures. Third, we do not account for model error in the input data. Incorporation of model error into machine learning models is not trivial but future work will attempt to account for these errors as well as dynamical uncertainty and lagged uncertainty. Fourth, the machine learning models presented in this study did not explicitly consider temporal relationships. We believe that incorporation of the temporal dynamics in the machine learning could help regularize the estimation procedure by coupling observations across time while simultaneously providing useful scientific information about the temporal dynamics of the MLD in relation to the surface variables. Fifth, future work can explore the sensitivity of the predictive models to other input observations. Perhaps the most promising variable to consider would be the near‐surface wind speed, which can be obtained from satellite scatterometer observations. Finally, given further advances in the previous action items, we hope that this methodology can be used in conjunction with, or compete with, ocean data assimilation reanalyses. In addition to the continued methodological research that follows from this study, we believe that, given additional data in the future, this methodology can be used to answer more detailed questions about the variability of the MLD and scientific oceanographic research questions that require fine resolution gridded MLD estimates. This future work should include efforts to fully explain and understand the physical basis for the relationships between SST, SSS, SSH, and MLD that are quantified and leveraged for MLD prediction but not fully explained in this study.

## Figures and Tables

**Figure A1:**
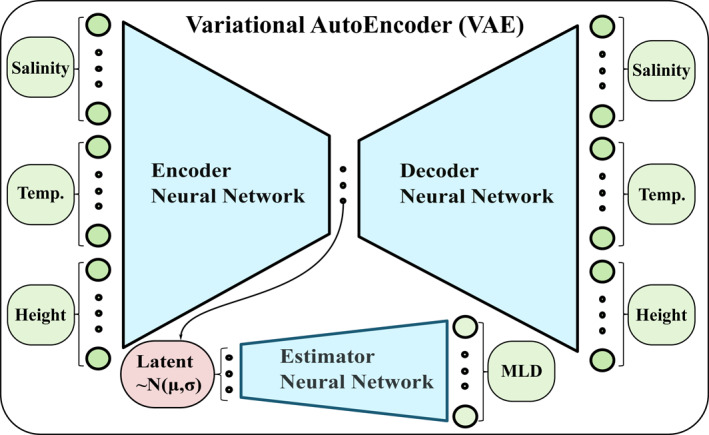
A schematic of the modified variational auto‐encoder (VAE). On the left hand side, the SSS, SST, and SSH inputs are fed into a neural network, called the Encoder network, that produces an output of smaller dimension—the latent space. On the right hand side, a second neural network, called the Decoder network, takes the latent variables as inputs and attempts to reproduce the original SSS, SST, and SSH inputs. In our implementation of the VAE, there is a third neural network that takes the latent space as inputs and outputs an estimate of the mixed layer depth. Training is informed by the decoder and estimator networks losses. For a full description of the training procedure for a typical VAE, see Kingma and Welling (2014).

**Figure B1:**
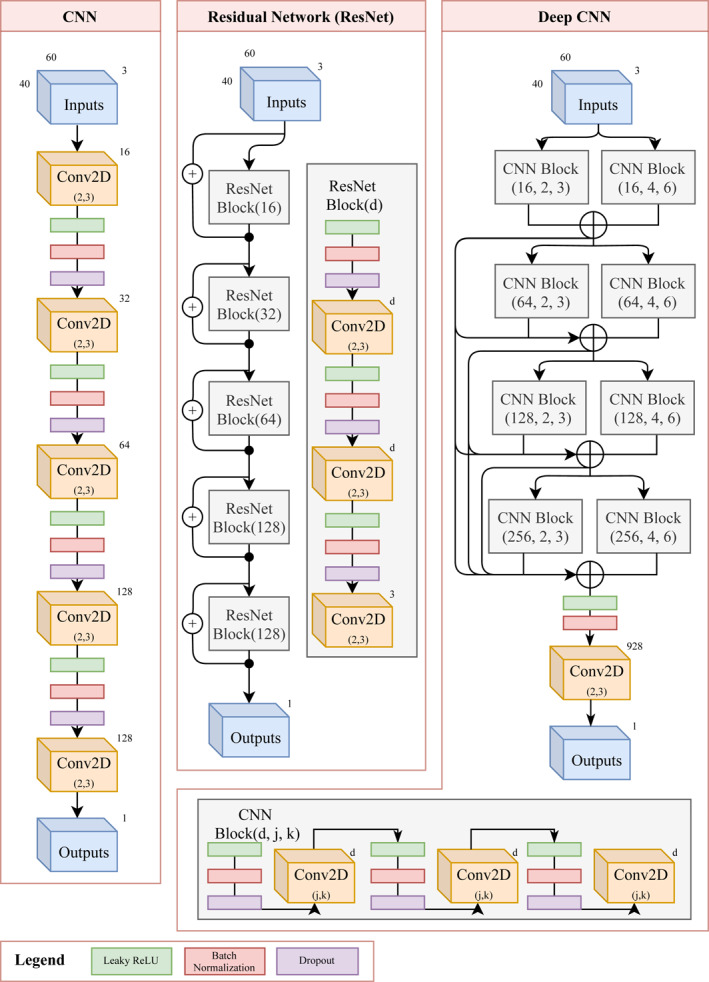
Architecture map of the three leading types of machine learning models used in this manuscript: “CNN,” “ResNet,” and “Deep CNN.” See the text for details.

## Data Availability

Code and examples for this project can be found at https://github.com/NCAR/ml-ocean-bl and https://doi.org/10.5281/zenodo.4441098. Argo‐based mixed layer depth data (Whitt et al., 2020) can be accessed at https://doi.org/10.5281/zenodo.4291175. Preprocessed surface and mixed layer data and model outputs (Foster et al., 2020) can be accessed at https://www.doi.org/10.5281/zenodo.4421752.

## Appendix A Probabilistic Machine Learning Models

### A1 Dropout

The simplest technique to introduce uncertainty estimates into a neural network is to implement Dropout (Hinton et al., 2012; Srivastava et al., 2014). Acting as a layer of the network, Dropout randomly sets inputs to zero at a particular rate and scales the rest of the inputs by 1/(1 − rate). Mathematically,

(A1)
$$f^{\left(i\right)}\left(x\right)=\frac{1}{1-p}M⊙a\left(x^{T}W_{i}+b_{i}\right),\;M_{j}∼\text{Bernoulli}\left(p\right),$$

where ⊙ means element‐wise multiplication. Each run of the model then has a different combination of weights that are set to zero. While originally this technique was used to reduce overfitting, it can also be viewed through a Bayesian probabilistic lens (Maeda, 2014). Running the model multiple times creates an ensemble that can be used to calculate moments of the output distribution, and, in particular, Σ and *μ*. It has been shown that the expected distribution from a neural network utilizing Dropout forms a Gaussian mixture distribution (Gal & Ghahramani, 2016). Therefore, there is some reason to believe that the regularity of the data distribution dictates how useful Dropout can be in uncertainty quantification.

### A2 Variational Networks

The next simplest probabilistic technique, what we call the Variational Artificial Neural Network (VANN), also known as a heteroscedastic network, is to parameterize the output of the neural network according to some distribution. For a Gaussian distribution, for example, the output of *f* is a stacked vector of the mean and covariance estimates,

(A2)
f(SSS,SST,SSH;θ)=[μ;vec(Σ)],

where vec(*Σ*) is the flattened covariance matrix, such that *d* ∼ *N*(*μ*, *Σ*). This technique is relatively easy to implement with care needed to ensure that constraints on the parameters are enforced. Typically, a Bayesian framework would then impose prior probability distributions onto *μ* and *Σ*. In particular, in addition to the Gaussian likelihood, it is common to impose a Gamma or LKJ ‐ uniform over the space of covariance matrices ‐ prior on the covariance to prevent unnecessary shrinkage. In a feedforward neural network, this parameterization increases the number of outputs and hence the overall total number of parameters. If the number of grid points of *d*(**x**) is *M* then a full covariance matrix would require *M*(*M* + 1)/2 parameters and the corresponding number of parameters required in the neural network makes it computationally prohibitive as *k* grows large. To limit the computational cost, we make a diagonal assumption about the covariance to reduce the number of parameters at the expense of losing covariance information between MLD values at different grid points. Parameterization of the data distribution is not always possible if a good approximation or transformation to an appropriate probability distribution is not known and the effectiveness of this technique is reflection of the quality of that assumption.

### A3 Variational Auto‐Encoders

Another method that we test is the variational auto‐encoder (VAE) (Kingma & Welling, 2014). A typical VAE consists of two neural networks: an encoder that projects the inputs into a lower‐dimensional latent space, parameterized by a probability distribution, and a decoder that inverts this projection and produces the original input. The loss between the decoder's output and the original system drives the learning process. A VAE supposes a prior distribution over the latent variable *z*, *p*(*z*), that, along with the decoder network that induces a conditional likelihood distribution *p*(SSS, SST, SSH|*z*; *θ*), forms a posterior distribution,

p(z|SSS,SST,SSH;θ)∝p(z)p(SSS,SST,SSH|z;θ).

This posterior distribution is typically intractable and thus replaced by a variational approximation *q*(*z*|SSS, SST, SSH; *θ*). This approximation includes a parameterization of the prior and likelihood distributions, typically Gaussian distributions with parameters that are learned in the encoder network. In our design we also use a Gaussian distribution in the latent space, and, as demonstrated in Figure A1, we couple this network with a third dense network, which we call the estimator, that transforms the latent space into an estimate of the MLD associated with the surface salinity, temperature, and sea height anomaly encoder inputs.

While the prior and likelihood distributions in a VAE are specified as Gaussian, the distribution of the output of the estimator network, that is, the MLD outputs, is not parameterized. While the difference between the MLD estimates and the MLD observations is modeled as a Gaussian process regardless of neural network architecture, the possible benefit of our chosen VAE approach is that it can produce theoretically arbitrary probability distribution *p*(*d*|SSS, SST, SSH; *θ*). Another theoretical benefit to this approach is that, since the neural network can learn an efficient lower‐dimensional representation of the inputs that capture dominant patterns, the estimator might be better able to generalize and to be less sensitive to small perturbations and noise in the inputs.

### A4 Deep Ensembles

The final method for uncertainty quantification that we consider in this study is called “Deep Ensembles” in the literature (see Lakshminarayanan et al., 2017). In short, we create an ensemble of neural networks by initializing each with different weights and training them individually (the order of the training data set is shuffled for each ensemble member). During testing, a random ensemble member is drawn and queried to produce a result. Sample based techniques can then be computed from the resulting ensemble of predictions. This technique naturally increases the computational cost of a traditional neural network architecture by the number of ensemble members and may be impractical for large systems. Note that uncertainty is not a learned trait in this approach, but inherited from the ability of the neural network architecture to transfer input uncertainty into output uncertainty. Therefore, the utility of this method depends heavily on the application details. Recent work by Weyn et al. (2019) and Weyn et al. (2021) suggest that this technique may be useful in weather modeling.

## Appendix B Machine Learning Model Architectures

### B.1

During this project we tested numerous machine learning models, architectures, and hyperparameter sets for each model type, many of which are not presented in this manuscript. During the course of evaluating each of the models on the test data set, we narrowed in on a handful of good performing models of a varying degree of parameterization and size. Because of the near‐infinite set of possible configurations, our search is not, and perhaps cannot be, exhaustive. Furthermore, in the future when “sufficient” data becomes available, the relative performance of machine learning models is subject to change and “deeper” machine learning models may become more competitive.

In combination with Table 1, which details the nomenclature and size of the models that we present, Figure B1 shows the architecture design of the three main types of machine learning models presented in the main text: the “CNN” architecture, which is also the basic design for the “Dropout” (the difference is in Monte Carlo dropout applied during testing and validation instead of only during training), “VLCNN,” and “VCNN” models; the “ResNet” architecture; and the “Deep CNN” architecture, which is also used for the “Deep VCNN” models.

The “CNN” architecture has four sets of a pattern of layers that consists of a 2D convolutional layer, whose kernel size—(2,3) for the EPO, (2,4) for the SIO—is shown in the figure (each convolution is implemented with padding to keep input and output sizes consistent), followed by the “Leaky ReLU” activation function, a “Batch Normalization” layer, and “Dropout” layer with dropout probability of 0.1. Each convolution layer has an associated number of layers that it produces, called filters (representing separate convolution kernels, sets of parameters, that should manifest into visualization of different features sets in the input data). The number of features, or filters, produced by each convolution layer is written in the figure directly northwest of the convolution layer. The final output convolution layer produces one filter for a deterministic output (referred to as “CNN” in the text), or, two outputs for a mean and variance map (this configuration is referred to as “VCNN”). The “VLCNN” model has the same “CNN” architecture with one filter output and a variance produced separately by a linear affine map of the inputs. Technically, the softplus function is applied to the outputs of these models to ensure positive‐definiteness of the variance. See the TensorFlow documentation for further details about the layer mechanics (Abadi et al., 2016).

The “ResNet” architecture is typical of other Residual Neural Networks (see He et al., 2016), with series of blocks whose outputs are summed with the input of the block to produce the input into the next block. The output of each ResNet block must have the same number of filters as in the input data, three (SSS, SST, and SSH), in order for the additive function to be well‐defined. This additive nature is known to help avoid gradient propagation issues in deep machine learning models. The structure of each block is similar to the “CNN” architecture, but with activation preceding the convolution layer. We found this ordering of activation functions to be slightly superior to other combination of layer order.

Finally, the “Deep CNN” architecture is inspired from a combination of ResNet and U‐Net (see Ronneberger et al., 2015) architecture designs, where intermediate layers that are theorized to capture features of varying spatial scales and physical properties are concatenated (denoted by the simple *⊕* in the figure) in series. The “Deep CNN” is designed to promote the sharing of information between scales and to avoid vanishing gradient problems during training. Through the course of the series of CNN blocks, the effective size of the Receptive Field, the size of the region in the original image that produces a data‐point in the output filter, grows and increasingly represents progressively larger spatial features. Concatenating earlier and later layers ensures that the final convolutional layer has direct access to small and large scale features for predicting MLD. The architecture uses combinations of CNN blocks, shown in the bottom of the figure, that act similar to the ResNet block, a series of convolutional layers preceded by activation functions, but are not constrained in output filter size. In the “Deep CNN,” we concatenate CNN blocks of progressively larger output filter size (parameterized by “*d*” in the figure).

## Appendix C Gaussian Process Regression

### C.1

Gaussian Process Regression is closely related to the somewhat more general Optimal Interpolation and Kriging frameworks. For a more detailed history and exposition, see Cressie (1993). A Gaussian process is any collection of random variables for which any finite number have a joint Gaussian distribution and, as a result, is completely determined by a mean and covariance function (Rasmussen & Williams, 2006). Given a set of (two‐dimensional) observation locations $x={\left(x_{1},\ldots ,x_{M}\right)}^{T}$, we define the mean function *m*(**x**) and the covariance function *k*(**x**, **x**′) of the process *d*(**x**) as

(C1)
m(x)=E[d(x)]

(C2)
$$k\left(x,x^{\prime }\right)=E[\left(m\left(x\right)-d\left(x\right))(m\left(x^{\prime }\right)-d\left(x^{\prime }\right)\right)]$$

where **x**′ is another (or possibly identical) set of input locations.

Typically the mean function is set to zero and covariance function is parameterized according to some kernel function. Various kernel functions impart different types of regularity (differentiability): the exponential kernel leads to non‐differentiable outputs, the Matern Class of kernels have a regularity parameter, and the squared exponential kernel leads to smooth outputs. In our study, the squared exponential kernel,

(C3)
$$k\left(x,x^{\prime }\right)=\alpha e^{-\frac{1}{2\ell }\|x-x^{\prime }{\|}^{2}}+\beta$$

where *α* and *ℓ* are hyperparameters that control the amplitude and length‐scale of the corresponding covariance structure, was chosen because of its marginally better performance and efficiency compared to Matern class kernels. We train our Gaussian process hyperparameters by optimizing according to the Gaussian process prior probability distribution over the training observation points **x**,

(C4)
$$\ln p\left(\alpha ,\ell ,\beta |d\right)=-\frac{1}{2}d^{T}K{\left(x,x\right)}^{-1}d-\frac{1}{2}\ln|K\left(x,x\right)|-\frac{M}{2}\ln2\pi ,$$

where the covariance matrix has entries *K*_*i*,*j*_(**x**, **x**) = *k*(*x*_*i*_, *x*_*j*_). To regularize the optimization process and ensure positivity of *α*, *ℓ*, and *β*, priors are occasionally placed on the hyperparameters in a Bayesian fashion. In our study, this type of implementation had minimal impact on the optimized values. In circumstances where either computational considerations are not a concern or available training data is limited, it is also possible to optimize the hyperparameters by cross‐validating and minimizing the conditional likelihood distribution, for details see Rasmussen and Williams (2006). The variance hyperparameter *β* can, in general, be made anisotropic at the expense of increasing the total number of hyperparameters, but we do not consider such options in this study.

During the training of the neural network, that is, while optimizing the parameters in *f* via Equation 3 using backpropagation on training data from a given week, the Gaussian process hyperparameters must be re‐optimized according to Equation C4 because the Gaussian process parameterization depends on the Argo profile locations (and model covariance Σ, if available) which generally vary from one training week to the next.

Once the Gaussian process has been optimized using function values (**x**, *d*), we can perform inference at the Argo spatial locations **x**_*o*_ to obtain estimates of *d*_*o*_. The inference procedure follows Equation 2 with *L* and *V* given by the equations

(C5)
$$L=k\left(x_{o},x\right){\left(k\left(x,x\right)+\Sigma \right)}^{-1}$$

(C6)
$$V=k\left(x_{o},x_{o}\right)-k\left(x_{o},x\right){\left(k\left(x,x\right)+\Sigma \right)}^{-1}k(x,x_{o}).$$

Thus, the trained kernel function is independent of time and depends only on distance ‖**x** − **x**′‖ not location **x** or time, but *L* and *V* depend on location and time because Σ depends on location **x** and the particular points chosen for estimation **x**_**o**_ (e.g., the Argo profiles locations) vary with time.
